# Imaging Mass Spectrometry and Proteome Analysis of Marek’s Disease Virus-Induced Tumors

**DOI:** 10.1128/mSphere.00569-18

**Published:** 2019-01-16

**Authors:** V. I. Pauker, L. D. Bertzbach, A. Hohmann, A. Kheimar, J. P. Teifke, T. C. Mettenleiter, A. Karger, B. B. Kaufer

**Affiliations:** a Institute of Molecular Virology and Cell Biology, Friedrich-Loeffler-Institut, Greifswald-Insel Riems, Germany; b Institute of Virology, Freie Universität Berlin, Berlin, Germany; c Department of Poultry Diseases, Faculty of Veterinary Medicine, Sohag University, Sohag, Egypt; d Department of Experimental Animal Facilities and Biorisk Management, Friedrich-Loeffler-Institut, Greifswald-Insel Riems, Germany; Northwestern University Feinberg School of Medicine

**Keywords:** Marek’s disease virus, imaging mass spectrometry, lymphoma, noncontact laser capture microdissection, proteome, tumor, tumor markers

## Abstract

The highly oncogenic alphaherpesvirus Marek’s disease virus (MDV) causes immense economic losses in the poultry industry. MDV induces a variety of symptoms in infected chickens, including neurological disorders and immunosuppression. Most notably, MDV induces transformation of lymphocytes, leading to T cell lymphomas in visceral organs with a mortality of up to 100%. While several factors involved in MDV tumorigenesis have been identified, the transformation process and tumor composition remain poorly understood. Here we developed an imaging mass spectrometry (IMS) approach that allows sensitive visualization of MDV-induced lymphoma with a specific mass profile and precise differentiation from the surrounding tissue. To identify potential tumor markers in tumors derived from a very virulent wild-type virus and a telomerase RNA-deficient mutant, we performed laser capture microdissection (LCM) and thereby obtained tumor samples with no or minimal contamination from surrounding nontumor tissue. The proteomes of the LCM samples were subsequently analyzed by quantitative mass spectrometry based on stable isotope labeling. Several proteins, like interferon gamma-inducible protein 30 and a 70-kDa heat shock protein, were identified that are differentially expressed in tumor tissue compared to surrounding tissue and naive T cells. Taken together, our results demonstrate for the first time that MDV-induced tumors can be visualized using IMS, and we identified potential MDV tumor markers by analyzing the proteomes of virus-induced tumors.

**IMPORTANCE** Marek’s disease virus (MDV) is an oncogenic alphaherpesvirus that infects chickens and causes the most frequent clinically diagnosed cancer in the animal kingdom. Not only is MDV an important pathogen that threatens the poultry industry but it is also used as a natural virus-host model for herpesvirus-induced tumor formation. In order to visualize MDV-induced lymphoma and to identify potential biomarkers in an unbiased approach, we performed imaging mass spectrometry (IMS) and noncontact laser capture microdissection. This study provides a first description of the visualization of MDV-induced tumors by IMS that could be applied also for diagnostic purposes. In addition, we identified and validated potential biomarkers for MDV-induced tumors that could provide the basis for future research on pathogenesis and tumorigenesis of this malignancy.

## Abstract (precis)

Marek’s disease virus (MDV) is an oncogenic alphaherpesvirus that infects chickens and causes the most frequent clinically diagnosed cancer in the animal kingdom. Not only is MDV an important pathogen that threatens the poultry industry but it is also used as a natural virus-host model for herpesvirus-induced tumor formation. In order to visualize MDV-induced lymphoma and to identify potential biomarkers in an unbiased approach, we performed imaging mass spectrometry (IMS) and noncontact laser capture microdissection. This study provides a first description of the visualization of MDV-induced tumors by IMS that could be applied also for diagnostic purposes. In addition, we identified and validated potential biomarkers for MDV-induced tumors that could provide the basis for future research on pathogenesis and tumorigenesis of this malignancy.

## INTRODUCTION

Marek’s disease (MD) is caused by the oncogenic *Gallid alphaherpesvirus 2*, also known as Marek’s disease virus (MDV). It is characterized by various clinical symptoms, including neurological disorders, immunosuppression, and most notably tumors in visceral organs (1). Remarkably, MD causes high economic losses in the poultry industry worldwide and is used as a natural virus-host small-animal model for herpesvirus-induced cancers (2–4). MDV infection can cause mortality of up to 100% in susceptible chickens; however, the severity of disease and mortality are dependent on the genetic background of the host, vaccination status, and virulence of the virus strain (5). Upon infection of the host, MDV efficiently spreads to lymphoid organs and replicates in B and T cells. T cells are the target for the establishment of latency and transformation (6), while B cells have been recently shown to be dispensable for MDV pathogenesis (7). Most latently infected and transformed cells are CD4^+^ T cells that rapidly replicate, resulting in the clinical signs and deadly lymphomas (8). These MDV-induced tumors efficiently develop in infected animals and can be observed as early as 3 to 4 weeks after infection (2). Several viral factors contribute to this rapid transformation as reviewed recently (9); these viral factors include the major oncoprotein Meq (2, 10, 11), the viral chemokine vIL-8 (12–14), MDV-encoded microRNAs (15–17), viral telomeric repeats (TMRs) (18–21), and a virus-encoded telomerase RNA (vTR) (22–25). For example, deletion of vTR severely impaired disease progression, tumor formation, and dissemination, while lytic replication was not affected (23, 26–28). Even though recent work has shed light on the role of vTR in MDV-induced tumor formation, many questions including whether tumor composition and markers differ in the absence of vTR, still remain unanswered. Recent advances in imaging mass spectrometry (IMS) techniques made it possible to link histological structures directly to mass spectrometric data (29). IMS has been used to visualize the distribution of a variety of biomolecules, including proteins with a wide molecular mass range, making it an extremely versatile tool. In the context of tumor biology, IMS allowed the identification of tumor markers from biopsy tissue sections (30). The proteins present in these tumor samples can be identified by additional mass spectrometry (MS) techniques. Biomarker candidates are subsequently validated by independent methods such as RT-qPCR. Until now, this approach had not been applied to MDV-induced tumors, and therefore, we lack reliable tumor markers. In this study, we applied IMS to MDV-induced lymphomas for the first time. We identified specific protein masses that were present in the tumor, but not the surrounding tissue. This allowed accurate visualization of tumors within healthy tissue and was furthermore confirmed by histochemistry. To identify potential tumor markers, we performed laser capture microdissection (LCM) on these tumor tissues. Several potential tumor markers were identified and confirmed by RT-qPCR.

## RESULTS

### MALDI imaging of MDV-induced lymphomas.

**FIG 1:**
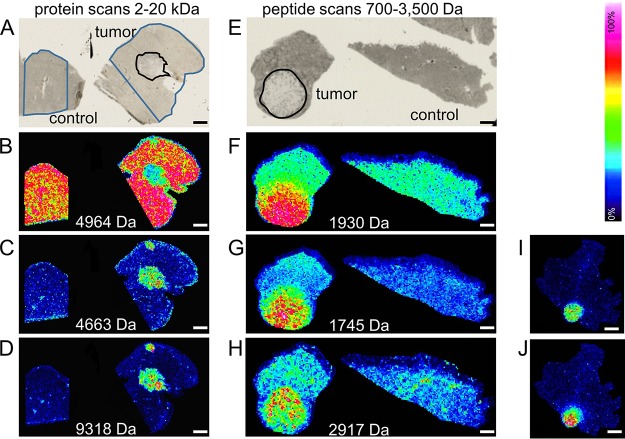
Protein and peptide IMS analysis of MDV-induced lymphomas. (A to D) Cryosections were prepared from the liver of a MDV-infected chicken, and an IMS protein scan was performed in the mass range between 2 and 20 kDa. The selected masses represent a marker for liver tissue (4,964 Da in panel B) and two for MDV-induced T cell tumors (4,663 Da and 9,318 Da in panels C and D, respectively). The remaining panels, panels E to J, show FFPE chicken liver (E to H) and chicken skeletal muscle (I and J) sections scanned in the typical peptide range (700 to 3,500 Da) after trypsin digestion of the sections. Panels A and E show HE-stained sections with or without (control) tumor; the regions that were measured by IMS are outlined in blue, and the tumor regions are outlined in black. IMS scans based on negative (B) and positive (C to D and F to H) tumor markers are shown. Panels I and J show the distributions of two tumor-specific masses (1,745 and 2,917 Da, respectively) that were identified in liver and skeletal muscle tumors from different animals. Intensities are rainbow color coded from 0% (black) to 100% (white) relative intensity according to the color code bar on the right. The represented masses are given for each panel. Bars, 1 mm.

**FIG 2:**
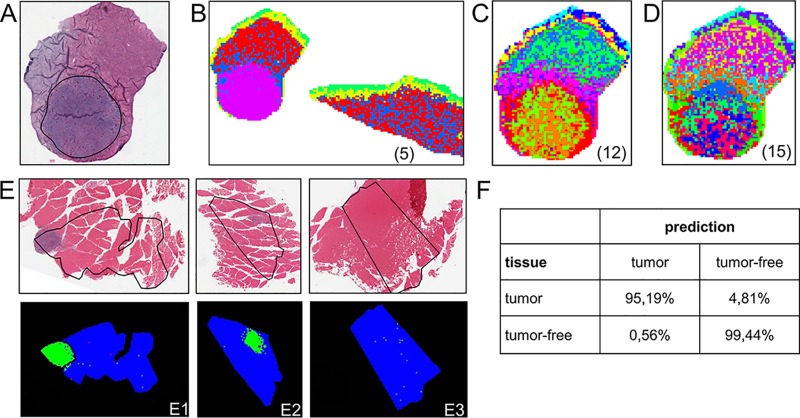
Statistical evaluation of MDV lymphoma peptide spectra. (A to D) Cluster analysis of spectra from a FFPE chicken liver section. (A) HE stain, the contour highlights the tumor region. (B) Joint cluster analysis of spectra from the section shown in panel A and a tumor-free control section with a given number of five clusters. Red and blue regions indicate tumor-free regions, which are clearly distinguished from the tumor appearing in magenta. Allowance of higher numbers of clusters as given in the parentheses in panels C and D resulted in a more fine-grained pattern. The margins of the tumor form a distinct red cluster in panel C, and the central region of the tumor shows microheterogeneity in, e.g., the green, dark blue, and red clusters in panel D. The color coding applies to each panel separately. The analysis was performed with in-house scripts using the statistical programming language R (63). (E1 to E3) Statistical models based on tumor-specific mass patterns of the chicken breast muscle tissue sample E1 were calculated using ClinProTools (Bruker) software. Spectra from the region outlined by the blue contour of the micrograph (E1) were used as training data for tumor (outlined in black) and tumor-free (outside the black outline) tissue. Models were then used to classify spectra from tissue sections of different animals that contained a tumor (E2) or were tumor-free controls (E3). Cross-validation of the models using the training data (E1) was correct for >99% of the data points. The regions predicted as tumor or tumor-free in the test sections E2 and E3 are shown in green and blue, respectively, and corresponded very well to the histological assessment of the sections (see Fig. S2 in the supplemental material). (F) The confusion matrix gives the prediction results of spectra from E2 and E3 in raster spots. In the tumor section, >95% of the area was correctly identified (*n* = 416), and prediction of the tumor-free region was correct for >99% (*n* = 3,036) showing that detection of MDV-induced tumors by IMS is feasible and exhibited high sensitivity and specificity.

To determine whether IMS is applicable for the detection of MDV-induced lymphomas, we analyzed sections of organs from MDV-infected chickens (Fig. 1). Lymphoma-specific mass signatures were readily identified in liver samples based on intact proteins with a mass range between 2,000 and 20,000 Da. Furthermore, we analyzed the peptides after proteolytic digestion of the tissue sections and scanned typical “peptide mass ranges” between 700 and 3,500 Da (Fig. 1F to J). Intriguingly, several identical lymphoma-specific masses were reliably detected in lymphomatous lesions that had developed in different organs obtained from different chickens (Fig. 1). Statistical evaluation of peptide spectra by cluster analysis revealed highly discriminative marker mass sets. Depending on the number of given clusters, the entire tumor was mapped as a single cluster, or some degree of differentiation within the tumor was revealed (Fig. 2), defining a region with specific expression profiles along the border of the tumor (Fig. 2C) or small islets with identical mass signatures that were interspersed within the tumor area (Fig. 2C and D). These results indicated that protein expression patterns for example at the margins of the tumor might differ from areas that are more central (Fig. 2B and C).

### Laser-dissected tissue sections and mass spectrometry.

To investigate the tumor tissue proteome in greater detail and to obtain potential tumor markers, we performed laser capture microdissection (LCM) on MDV-induced tumors and quantified the protein content by mass spectrometry. MDV-induced lymphomas are solid and consist of a mixture of pleomorphic lymphocytes, including malignantly transformed T cells, reactive B and T cells, and also macrophages, that differ unequivocally from nonneoplastic tissue such as liver lobules (Fig. 1A), allowing the precise differentiation from surrounding nontransformed liver tissue. Samples were extracted from MDV-induced tumors (RB-1B strain) by LCM and lysed, and the protein content was quantified (10 to 15 µg of protein per sample). In addition, we analyzed tumors induced by a mutant MDV that lacks the telomerase RNA gene vTR (RB1B-ΔvTR) and that were more compact and mostly consisted of lymphocytes (see Fig. S1 in the supplemental material). For negative controls, we used primary nontransformed chicken T cells, the target cells of MDV transformation, as well as unaffected liver tissue in order to reduce false-positive results resulting from any remaining contamination. Two independent samples were isotope labeled by dimethylation, fractionated, and analyzed by LC-MALDI TOF/TOF MS. In total, about 1,000 proteins were reliably identified when comparing wild-type RB-1B (959 and 841 proteins) or RB1B-ΔvTR (1,314 or 919) tumors with primary chicken T cells.

10.1128/mSphere.00569-18.1 FIG S1 Quantitation of cell types in different tumor samples with the Halo software. Six distinct areas of three different tumors per tumor type (RB1B or RB1B-ΔvTR derived) and in healthy liver tissue were stained (HE and CD3) and analyzed. The percentages of CD3^+^ and CD3^−^ lymphocytes, connective tissue, and liver tissue were calculated. Download FIG S1, TIF file, 0.1 MB. Copyright © 2019 Pauker et al. 2019 Pauker et al. This content is distributed under the terms of the Creative Commons Attribution 4.0 International license.

10.1128/mSphere.00569-18.2 FIG S2 Halo evaluation of MD tumors. Representative tissue sample from tumorous liver with MDV-induced lymphoma. CD3^+^ T cells (red), CD3^−^ T cells (green), hepatocytes (cyan blue), connective tissue (yellow), and blank space (purple) are indicated. CD3^+^ T cells clearly dominate the tumor area and can be differentiated from hepatocytes. Download FIG S2, TIF file, 7.2 MB. Copyright © 2019 Pauker et al. 2019 Pauker et al. This content is distributed under the terms of the Creative Commons Attribution 4.0 International license.

### Identification and confirmation of potential tumor markers.

Next, we set out to determine differentially expressed proteins between tumor samples and naive T cells by quantitative MS based on introduced isotope labeling. In total, 19 promising potential transformation markers could be identified (Table 1 and Table S1) which were also differentially expressed when tumor samples were compared to healthy liver tissue samples. Eight proteins were upregulated and eleven were downregulated in MDV-induced tumors (wild-type RB-1B and RB1B-ΔvTR) compared to primary T cells and healthy liver controls. To confirm the potential transformation markers identified through our proteomic analysis, we assessed the mRNA levels of several randomly selected transformation markers in different tumor samples. RNA was isolated from laser-dissected material, healthy nontransformed tissue, and naive T cells and analyzed by RT-qPCR. All tested potential transformation markers could be confirmed by RT-qPCR (Table 1), with TAP1 as the only exception. IFI30, OASL, and a HSP70 were found to be upregulated in tumor samples as observed in the proteomic analysis (Table 1). Similarly, LBR, GSTT1L, RCC2, FYB, and H2AJF were downregulated on both the mRNA and protein level (Table 1). Taken together, we identified several potential tumor makers that in most cases could be confirmed by RT-qPCR.

**TABLE 1:** Potential transformation markers

Ensembl accession no.	Protein (abbreviation)	Fold change^*a*^ by:	
		MS	RT-qPCR
ENSGALP00000005345	Interferon gamma-inducible protein 30 (IFI30)	3.83	3.56
ENSGALP00000041758	Transporter 1 ATP-binding cassette subfamily B (TAP1)	3.26	0.78
ENSGALP00000010210	Leukocyte cell-derived chemotaxin 2 (LECT2)	2.82	
ENSGALP00000016536	Heat shock 70-kDa protein 4-like (HSP70)	2.53	1.42
ENSGALP00000028664	2'-5′-Oligoadenylate synthetase-like (OASL)	2.49	2.10
ENSGALP00000039235	Cold shock domain containing E1 (CSDE1)	2.39	
ENSGALP00000013029	Splicing factor 3b subunit 1 (SF3B1)	2.29	
ENSGALP00000042479	Stress-induced phosphoprotein 1 (STIP1)	2.23	
ENSGALP00000011961	Phosphatidylethanolamine binding protein 1 (PEBP1)	0.47	
ENSGALP00000016363	Heterochromatin protein 1 binding protein 3 (HP1BP3)	0.47	
ENSGALP00000015128	Lamin B receptor (LBR)	0.42	0.20
ENSGALP00000010358	p21 protein (Cdc42/Rac)-activated kinase 2 (PAK2)	0.41	
ENSGALP00000033650	FYN binding protein (FYB)	0.39	0.44
ENSGALP00000003584	H3 histone family 3B (H3F3B)	0.35	
ENSGALP00000039872	Regulator of chromosome condensation 2 (RCC2)	0.35	0.37
ENSGALP00000041526	Histone cluster 1 H4-VI germinal H4 mRNA (HIST1H4A)	0.34	
ENSGALP00000008341	Glutathione S-transferase theta 1-like (GSTT1L)	0.27	0.19
ENSGALP00000040653	H2A histone family member J (H2AFJ) mRNA	0.26	0.44
ENSGALP00000027541	High mobility group box 1 (HMGB1)	0.25	

a Fold changes by MS describe the relative expression of the proteins in the tumors in relation to the expression in T cells. Values of >2 indicate overexpression in the tumor, while values of <0.5 indicate stronger expression in the naive T cells. Mean values of the experiments with RB1B and RB1B-ΔvTR tumors are given. Fold change by RT-qPCR was calculated as 2^ΔCT^. For a list of identified peptides corresponding to the regulated proteins, see Table S1 in the supplemental material.

10.1128/mSphere.00569-18.3 TABLE S1 List of identified peptides associated with the differentially expressed proteins. m/z meas., measured *m*/*z* value; Dm/z [ppm], *m*/*z* deviation in ppm; Scores, Mascot peptide identification score; Range, range of peptide within protein; Accession, EMBL protein ID; Protein, EMBL protein description. Download Table S1, DOCX file, 0.04 MB. Copyright © 2019 Pauker et al. 2019 Pauker et al. This content is distributed under the terms of the Creative Commons Attribution 4.0 International license.

## DISCUSSION

The most prominent characteristic of MDV is the ability to transform T cells and cause lymphomas in infected animals. The onset of MDV-induced tumor development is very rapid and can occur within 3 to 4 weeks postinfection. MDV integrates its genome in latently infected and tumor cells, allowing maintenance of the viral genetic material in dividing cells (18, 19). The rapid replication that is mostly driven by the viral oncogenes ultimately leads to the fatal lymphoma formation in visceral organs (6). In this study, we developed an imaging mass spectrometry (IMS) approach to visualize MDV tumors and obtain a specific mass profile and accurate differentiation from the surrounding tissue. Intriguingly, differences regarding specific signatures at the margin and central area of the tumors were observed. Similar findings were recently described for the intratumoral microheterogeneity of myxoid sarcomas (31) and distinct expression profiles in the microenvironment of breast tumors (32), stressing the value of IMS as an “open-view” approach complementing the targeted analysis provided by classical histological techniques. Our data demonstrate that the derived signatures were specific and robust and may have diagnostic potential. In future studies, we will compare the signatures obtained to those of MD tumors caused by other MDV strains, ALV- and REV-induced tumors, and nonviral tumors in chickens. Moreover, we implemented a proteomic workflow with a strongly reduced risk of tissue contamination and, hence, increased sensitivity for biomarker identification. This workflow is based on laser capture microdissection (LCM) of MDV tumors and reference material, followed by proteome analysis based on quantitative mass spectrometry. After digestion of extracted proteins, the peptides from different samples were isotope coded by dimethylation, mixed at 1:1 ratios, and fractionated by off-gel isoelectric focusing (OG IEF) to reduce the complexity of the mixture and to improve resolution of the mass spectrometric analysis by LC-MALDI TOF/TOF MS. Proteomic analysis of microdissected MDV tumors compared to naive T cells and surrounding liver tissue samples identified only 19 potential transformation markers (Table 1), showing that the expression profiles of naive and MDV-transformed T cells are very similar. We assume that the low number of potential transformation markers that we have identified results mainly from the high purity of the analyzed tumor samples, which rules out false-positive results resulting from any contaminating nonneoplastic tissue (33–36). Most of the candidates could be confirmed by RT-qPCR, indicating that they may indeed play a role for transformation. With our workflow, we successfully addressed the issue of tumor sample contamination by surrounding tissue for the differential analysis of MD tumors, which is virtually unavoidable if macroscopically isolated tissues are analyzed. As a result of the proteomic analysis, eight proteins were seen upregulated, and eleven proteins were downregulated in MD tumors compared to T cells (Table 1). Surprisingly, we did not identify significant differences in the proteomes of RB1B- and RB1B-ΔvTR-induced tumors. This is very intriguing and suggests that the morphological differences are either due to changes in the RNA level or in proteins that are below the detection level. Several of the identified markers could also be verified on the transcript level by RT-qPCR (Table 1). Only one out of four markers, which were upregulated in the proteomic analysis, TAP1, could not be confirmed on the transcript level by RT-qPCR. However, it is well-known that mRNA and protein expression levels do not always correlate due to complex regulation of transcription, processing, and degradation of mRNA, translation, modification, and turnover of proteins, as well as the differences in the half-lives of mRNA and proteins (37–40). Hence, it is possible that while the transcriptome is fully adapted to a certain condition, the proteome has not fully responded yet (38). The strongest upregulation that we could observe applied to IFI30, which has diverse cellular functions. It maintains the redox state of the cell, influencing autophagy, cellular activation, and proliferation. It has been shown that IFI30 is involved in the processing of epitopes from viral glycoproteins, for example, gB from herpes simplex virus 1 (HSV-1) and that it plays a role in eliciting an immune response toward HSV-1 infection (41). In addition, cancer infiltrating antigen-presenting cells elicit MHC II antigen processing and presentation through IFI30, shaping an antitumor T cell strategy (42). IFI30 may also influence tumorigenesis through alteration of the redox state, and cell proliferation (41). Taken together, IFI30 upregulation hints toward a host antitumor response. Upregulation of the molecular chaperone protein HSP70 has been identified previously in MDV-induced tumor cells, and there is very strong evidence that the interaction of Meq and HSP70 is significant in MDV lymphomagenesis (43). Similarly, the proteins OASL and TAP1 were upregulated in MD tumors. While OASL is an interferon-induced protein that regulates the early phase of viral infection, proviral functions like enhancement of viral persistence are also associated with members of the OAS family (44). Previous experiments have shown that the interferon gamma-induced pathway is altered in a MDV-transformed chicken CD4^+^ T cell line, resembling activation of T cells (45). TAP1 is involved in MHC class I antigen presentation (46), and slight upregulation of TAP2 in the tips of feathers of MDV-infected chickens was reported previously (47). The upregulation of several immune response-associated proteins indicate an activation of T cells, which could enhance the proliferation of tumor cells. While several immune response-associated proteins were upregulated in MDV-induced lymphomas compared to T cells, proteins associated with transcription and nucleosome assembly were found to be downregulated, for example, RCC2 (48), H2AFJ (49), H3F3B (50), HP1BP3 (51), and LBR (52). This is consistent with microarray investigations of MDV transformation in chicken spleens where transcription-related processes were also found to be downregulated (53). Similarly, a proteomic analysis of MDV-infected chicken embryonic fibroblasts detected an increased presence of phosphoproteins in the nucleus, indicating an effect on transcription regulation (54). Furthermore, two of the downregulated proteins are associated with signaling pathways that regulate the cytoskeleton (FYB [55] and PAK2 [56]). Burgess et al. previously demonstrated that Hodgkin’s disease antigen (CD30) is upregulated in tumors induced by HPRS-16 or GA/22 in line 7_2_ and 6_1_ chickens as well as Ross broilers (57, 58). We did not observe this CD30 upregulation in tumors induced by the very virulent RB-1B strain upon infection of Valo SPF chickens, suggesting that the virus strain and chicken line might influence the upregulation of CD30. In this study, we established a pipeline for efficient IMS and LCM of MDV-induced tumors and could identify highly discriminative marker mass sets for these tumors. This confirms that IMS is an “open view” tool that is neither restricted to a defined analyte nor limited by the availability of antibodies, fluorescent chromophores, or nucleic acid probes. Furthermore, we successfully applied LC-MALDI TOF/TOF MS to analyze dimethyl-labeled OG IEF-fractionated peptides isolated from MDV lymphoma tissue compared to naive T cells and healthy liver tissue. Changes in host protein expression during the transformation process were analyzed. Further functional analyses are necessary to confirm a role of the identified markers during MDV-induced transformation.

## MATERIALS AND METHODS

### Ethics statement.

All animal work was conducted according to the national and international guidelines for humane use of animals. Animal experiments were approved by the Landesamt für Gesundheit und Soziales (LAGeSo) in Berlin, Germnay (approval number G0218/12 and T0245/14).

### Animals, cells, and tumors.

Specific-pathogen-free (SPF) white Leghorn eggs were obtained from Valo Biomedia (Osterholz-Scharmbeck, Germany), and chickens hatched in the animal facility of the Center for Infection Medicine, Berlin, Germany. Primary T cells were isolated from the thymuses of 6- to 11-week-old chickens by manual dissociation of the organ, followed by isolation of the cells by density gradient centrifugation as described previously (59, 60). T cells were pelleted and stored at −80°C prior to lysis. MDV-induced tumors were collected from birds infected with the BAC-derived very virulent wild-type RB1B virus (GenBank accession no. EF523390.1) (19, 61) and the RB1B-ΔvTR mutant (27). Briefly, birds were infected intra-abdominally with 4,000 PFU of the respective virus and were monitored for disease signs throughout the experiment. Birds with clinical signs were euthanized, and tumorous tissues were collected from chickens between 41 and 82 dpi. Tissues were fixed in formalin and stored at 4°C or snap-frozen in liquid nitrogen and stored at −80°C.

### MALDI imaging of MDV-induced lymphomas.

Relevant locations for IMS imaging were selected based on hematoxylin-and-eosin (HE)-stained tissue sections. IMS on formalin-fixed paraffin-embedded tissue (FFPE) was performed following a published protocol (62) and the guidelines of the manufacturer of the MS platform (Bruker). To this end, tissues were cut at 5 µm and mounted on a conductive indium tin oxide (ITO)-coated glass slides (catalog no. 8237001; Bruker Daltonik GmbH) to avoid charge build-up during the measurement of mass spectra. For peptide measurements, the FFPE tissue sections were stored at 56°C overnight and dewaxed by immersing the slide in xylene and rehydration in a graded ethanol series on the following day. The antigens were demasked by heating the tissue sections in 10 mM Tris buffer (pH 9.0) for 10 min at 110°C in a decloaking chamber (Biocare Medical). Subsequently, peptides were liberated by digestion with trypsin (Promega) on the tissue. Both the enzyme and later the matrix were applied by an automatic sprayer (ImagePrep; Bruker), following standard protocols suggested by the manufacturer, resulting in a homogenous matrix layer over the whole tissue section. α-Cyano-4-hydroxycinnamic acid (HCCA) was used as matrix for peptide and protein analysis. Spectra were acquired with an Ultraflex MALDI-TOF/TOF mass spectrometer. The software projects a grid of spots over the region of interest, and from every spot, a spectrum is acquired. Obtained spectra were normalized before images were exported. Colored graphs in Fig. 1 show the distribution and relative intensity of a specific mass in rainbow color code. Statistical models were calculated using the ClinProTools software (Bruker). For k-means cluster analysis, the peaklists of the spectra were exported to statistical software R (63) and processed with an in-house script. Peak alignments were calculated across all measured spectra using maximal bin sizes (mass tolerances) of 5,000 ppm with the R package caMassClass (version 1.9 [64]). Based on this matrix, k-means clustering was performed with a predefined number of clusters. For the graphic representation, images were reconstructed with the R package pixmap (version 04-11 [65]) using the clusters for color coding.

### Laser capture microdissection (LCM).

Frozen tumor tissues from two independent replicates per virus mutant were cut into 20-µm cryosections using a precooled HM 560 Cryostar cryostat (Microm International). Cryosections were fixed with ice-cold ethanol (70% for 1 min, followed by a dip in 100%) and dried prior to laser dissection for 1 h at RT in a mild vacuum (150 mm Hg). LCM was performed using the Palm MicroBeam system and the Palm RoboSoftware 4.5 (Zeiss) according to the manufacturer’s instructions. For each tumor, 10 identical rectangles of 2.5 mm^2^ each, equivalent to a total of approximately 10^4^ cells, and 10 such cuts from unaffected tissue were collected. Tissue cuts were dried by vacuum centrifugation, lysed in 30 µl lysis buffer (0.1 M Tris-HCl [pH 8.0], 0.1 M DTT, and 2% SDS), heated for 10 min at 95°C, and sonicated. For a control, 1 × 10^7^ chicken T cells were lysed in 100 µl lysis buffer. The supernatants were recovered for filter-aided sample preparation (FASP) digest as described previously (66). The protein contents of lysed samples were assessed by densitometry of Coomassie blue-stained SDS-PAGE gels with BSA standards (Merck) (67).

### Protein labeling and LC-MALDI TOF/TOF mass spectrometry.

Dimethyl labeling of peptides for the quantification of proteins in tumor tissue, unaffected liver tissue, and primary T cells was performed as described previously (68). Samples were desalted using Empore solid-phase extraction cartridges (3M) (66), mixed at 1:1 protein ratios, and fractionated by gel-free isoelectric focusing with an Agilent 3100 Offgel fractionator as described previously (69). Separated peptide fractions were further separated based on hydrophobicity on a LC column with the EASY-nLC II (Bruker) chromatographic system, spotted to a MALDI target (Proteineer fcII; Bruker), and analyzed in an UltrafleXtreme MALDI-TOF/TOF mass spectrometer (Bruker) as described previously (69). Peptide spectra were acquired in the *m/z* range 700 to 3,500 Da with a minimum signal-to-noise (S/N) ratio of 7. Proteins were identified on basis of the MS/MS with a Mascot server (version 2.4.1; Matrix Science [70]) and analyzed using ProteinScape software (version 3; Bruker). As sequence database, the Gallus gallus proteome downloaded from the ENSEMBL website (71) and the viral sequences were added to the FASTA file. Oxidation of methionine, acetylation of protein N′ termini, and dimethylation of lysine and peptide N′ termini (both isotopomeric forms) were set as variable modifications, whereas the carbamydomethylation of cysteine residues was set as a fixed modification. Up- and downregulated proteins were identified using ProteinScape (Bruker) and an in-house R script. Candidate protein markers showing at least twofold up- or downregulation were selected for confirmation by qPCR.

### Confirmation of transformation markers by RT-qPCR.

The RNA from cells and tissue sections was isolated with the RNeasy minikit (Qiagen) following the manufacturer’s instructions. Several randomly selected potential transformation markers were confirmed using one-step RT-qPCR using the qScript one-step SYBR Green qRT-PCR kit (Quantabio) in an CFX96 Touch real-time PCR detection system (Bio-Rad). Samples were measured in duplicates, and expression levels were calculated relative to expression of GAPDH, 28S rRNA, and β-actin (Table 2) using the 2^ΔCt^ method as described previously (72).

**TABLE 2:** Primers used in this study for quantitative PCR to confirm Gallus gallus genes as potential transformation markers

NCBI reference sequence	Gene name	Primer sequence^*a*^	Product size (bp)
NM_205041.1	2'-5′-Oligoadenylate synthetase like (OASL)	For: 5′-AGGTCCTGGTGAAGGACAGT-3′	145
		Rev: 5′-TCCAGCTCCTTGGTCTCGTA-3′	
	28S RNA	For: 5′-GGTATGGGCCCGACGCT-3′	144
		Rev: 5′-CCGATGCCGACGCTCAT-3′	
NM_205518.1	Actin, beta (ACTB)	For: 5′-GAGAAATTGTGCGTGACATCA-3′	152
		Rev: 5′-CCTGAACCTCTCATTGCCA-3′	
NM_001031451.1	FYN binding protein (FYB)	For: 5′-GCCCCAAAACGGAAGTCTTTGC-3′	256
		Rev: 5′-TGGGCTTGACATTTCTGGGCG-3′	
XM_001231970.4	Glutathione S-transferase theta 1-like (GSTT1L)	For: 5′-AACAGGCCAGGGTTGATGAG-3′	137
		Rev: 5′-AGCACCTTCCACTTTCTCCG-3′	
NM_204305.1	Glyceraldehyde-3-phosphate dehydrogenase (GAPDH)	For: 5′-AATGGCTTTCCGTGTGCCAACC-3′	223
		Rev: 5′-ATTCAGTGCAATGCCAGCACCC-3′	
NM_001030753.1	H2A histone family, member J (H2AFJ)	For: 5′-AGGCCAAGTCGCGTTCATC-3′	148
		Rev: 5′-ATCTCGGCCGTCAGGTACT-3′	
NM_001012576.1	Heat shock protein family A (Hsp70) member 4-like (HSPA4L)	For: 5′-TGGCGACAACTCCAAAGTGA-3′	129
		Rev: 5′-TCAGTATCCATCGCTGCGTC-3′	
XM_418246.5	Interferon, gamma-inducible protein 30 (IFI30)	For: 5′-CGCTCAGGAGAGGAATGTCT-3′	181
		Rev: 5′-GCAAGCCTTCAGATTCTTGG-3′	
NM_205342.1	Lamin B receptor (LBR)	For: 5′-GCAAACAAGATGACCCCAGC-3′	150
		Rev: 5′-GGCCTTCCACAACCTTTCCT-3′	
M75729.1	MDV 175-kDa protein (ICP4) gene	For: 5-TTTCTAGCAAGGAGCGACGC-3′	81
		Rev: 5-CTGACTTGCGCTTACGGGAA-3′	
XM_015296999.1	Regulator of chromosome condensation 2 (RCC2)	For: 5′-CTGGTTGTAGGCTTGGAGCA-3′	146
		Rev: 5′-TGAGGAAGGAGGGTGGGAAA-3′	
NM_001135968.1	Transporter 1, ATP-binding cassette, subfamily B (MDR/TAP) (TAP1)	For: 5′-ACGACTTCATCACTCGCCTGC-3′	280
		Rev: 5′-TCCAACACCACCACTCGTTGTG-3′	

a For, forward primer; Rev, reverse primer.

### Immunohistochemistry and quantitation of cell types.

To assess morphology and tumor morphology, 3-µm-thick FFPE sections were prepared and HE stained according to standardized procedures. By immunohistochemistry, T cells were detected using a polyclonal rabbit anti-human CD3 antibody (catalog no. A0452; Dako) (1:200) and the Vectastain Elite ABC HRP detection kit (Vector Laboratories). CD3-positive and -negative round cells, assumed to be B cells and macrophages, were quantified in three independent tumors induced by wild-type RB1B or RB1B-ΔvTR using the Halo imaging software (Indica Labs). Six randomly selected areas were quantified for each tumor, and healthy liver tissue sections were used as a control.
