# Supplementary figures and images for: Imaging Mass Spectrometry and Proteome Analysis of Marek’s Disease Virus-Induced Tumors
Source: mSphere. 2019 Jan 16;4(1):e00569-18. doi: 10.1128/mSphere.00569-18 (PMC6336081; doi:10.1128/mSphere.00569-18)

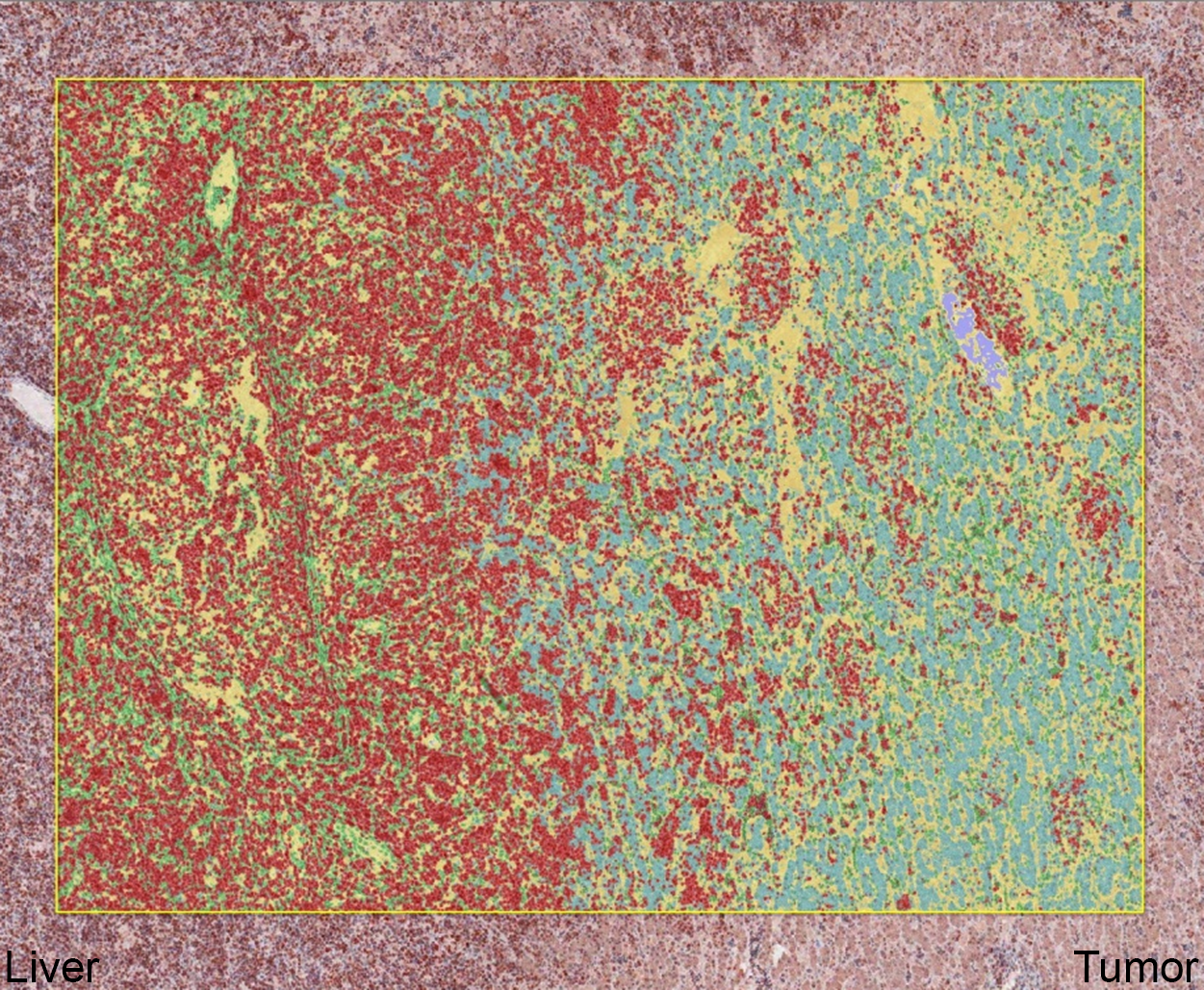

Supplement: FIG S2 [file mSphere.00569-18-sf002.tif]
