# Supplementary material for: Imaging Mass Spectrometry and Proteome Analysis of Marek’s Disease Virus-Induced Tumors
Source: mSphere. 2019 Jan 16;4(1):e00569-18. doi: 10.1128/mSphere.00569-18 (PMC6336081; doi:10.1128/mSphere.00569-18)
Supplement: TABLE S1 [file mSphere.00569-18-st001.docx]

| **m/z meas.** | **Dm/z [ppm]** | **Scores** | **Modifications** | **Range** | **Accession** | **Protein** |
| --- | --- | --- | --- | --- | --- | --- |
| 1728,9175 | -0,12 | 64.7 (M:64.7) | Dimethyl:2H(4): 1 | 214 - 228 | ENSGALP00000005345 | interferon gamma-inducible protein 30 - CGNC Symbol 2468 |
| 1724,8951 | 1,45 | 77.2 (M:77.2) | Dimethyl: 1 | 214 - 228 | ENSGALP00000005345 | interferon gamma-inducible protein 30 - CGNC Symbol 2468 |
| 1730,8943 | -2,02 | 73.1 (M:73.1) | Carbamidomethyl: 4; Dimethyl:2H(4): 1 | 175 - 190 | ENSGALP00000005345 | interferon gamma-inducible protein 30 - CGNC Symbol 2468 |
| 1726,874 | 0,75 | 35.1 (M:35.1) | Carbamidomethyl: 4; Dimethyl: 1 | 175 - 190 | ENSGALP00000005345 | interferon gamma-inducible protein 30 - CGNC Symbol 2468 |
| 1436,6481 | -5 | 42.8 (M:42.8) | Dimethyl: 1 | 209 - 221 | ENSGALP00000041758 | transporter 1 ATP-binding cassette sub-family B (MDR/TAP) - CGNC Symbol 53176 |
| 1440,671 | -6,56 | 61.4 (M:61.4) | Dimethyl:2H(4): 1 | 209 - 221 | ENSGALP00000041758 | transporter 1 ATP-binding cassette sub-family B (MDR/TAP) - CGNC Symbol 53176 |
| 1813,0117 | 2,11 | 34.5 (M:34.5) | Dimethyl: 1 | 549 - 564 | ENSGALP00000041758 | transporter 1 ATP-binding cassette sub-family B (MDR/TAP) - CGNC Symbol 53176 |
| 1817,0366 | 1,98 | 55.8 (M:55.8) | Dimethyl:2H(4): 1 | 549 - 564 | ENSGALP00000041758 | transporter 1 ATP-binding cassette sub-family B (MDR/TAP) - CGNC Symbol 53176 |
| 1598,961 | 2,7 | 76.3 (M:76.3) | Dimethyl:2H(4): 1 | 409 - 422 | ENSGALP00000041758 | transporter 1 ATP-binding cassette sub-family B (MDR/TAP) - CGNC Symbol 53176 |
| 2193,1159 | 3,43 | 56.3 (M:56.3) | Dimethyl:2H(4): 1 | 456 - 476 | ENSGALP00000041758 | transporter 1 ATP-binding cassette sub-family B (MDR/TAP) - CGNC Symbol 53176 |
| 878,5588 | 18,01 | 18.6 (M:18.6) | Dimethyl:2H(4): 1 | 522 - 529 | ENSGALP00000041758 | transporter 1 ATP-binding cassette sub-family B (MDR/TAP) - CGNC Symbol 53176 |
| 1132,4842 | 0,14 | 45.1 (M:45.1) | Carbamidomethyl: 3; Dimethyl: 1 | 51 - 60 | ENSGALP00000010210 | leukocyte cell derived chemotaxin 2 - CGNC Symbol 4754 |
| 1136,5107 | 1,43 | 60.8 (M:60.8) | Carbamidomethyl: 3; Dimethyl:2H(4): 1 | 51 - 60 | ENSGALP00000010210 | leukocyte cell derived chemotaxin 2 - CGNC Symbol 4754 |
| 807,4656 | -13,96 | 22.8 (M:22.8) | Dimethyl:2H(4): 1; Dimethyl: 6 | 137 - 142 | ENSGALP00000010210 | leukocyte cell derived chemotaxin 2 - CGNC Symbol 4754 |
| 807,4481 | -4,5 | 18.1 (M:18.1) | Dimethyl:2H(4): 1 | 287 - 292 | ENSGALP00000010210 | leukocyte cell derived chemotaxin 2 - CGNC Symbol 4754 |
| 1035,6132 | 0,01 | 23.3 (M:23.3) | Carbamidomethyl: 3; Dimethyl: 1 | 116 - 123 | ENSGALP00000010210 | leukocyte cell derived chemotaxin 2 - CGNC Symbol 4754 |
| 1039,6337 | -4,39 | 35.9 (M:35.9) | Carbamidomethyl: 3; Dimethyl:2H(4): 1 | 116 - 123 | ENSGALP00000010210 | leukocyte cell derived chemotaxin 2 - CGNC Symbol 4754 |
| 1179,5498 | -4,73 | 75.4 (M:75.4) | Carbamidomethyl: 3; Dimethyl:2H(4): 1 | 200 - 209 | ENSGALP00000010210 | leukocyte cell derived chemotaxin 2 - CGNC Symbol 4754 |
| 1175,5242 | -5,12 | 48.0 (M:48.0) | Carbamidomethyl: 3; Dimethyl: 1 | 200 - 209 | ENSGALP00000010210 | leukocyte cell derived chemotaxin 2 - CGNC Symbol 4754 |
| 1049,6282 | -0,57 | 24.7 (M:24.7) | Carbamidomethyl: 3; Dimethyl: 1 | 266 - 273 | ENSGALP00000010210 | leukocyte cell derived chemotaxin 2 - CGNC Symbol 4754 |
| 1730,8632 | 1,34 | 99.4 (M:99.4) | Dimethyl: 1 | 244 - 258 | ENSGALP00000010210 | leukocyte cell derived chemotaxin 2 - CGNC Symbol 4754 |
| 1734,9022 | 9,34 | 127.5 (M:127.5) | Dimethyl:2H(4): 1 | 244 - 258 | ENSGALP00000010210 | leukocyte cell derived chemotaxin 2 - CGNC Symbol 4754 |
| 1614,7477 | -7,24 | 39.4 (M:39.4) | Carbamidomethyl: 6; Dimethyl: 1 | 32 - 44 | ENSGALP00000010210 | leukocyte cell derived chemotaxin 2 - CGNC Symbol 4754 |
| 1231,6437 | 12,96 | 15.0 (M:15.0) | Dimethyl: 11 | 309 - 319 | ENSGALP00000010210 | leukocyte cell derived chemotaxin 2 - CGNC Symbol 4754 |
| 2449,2115 | 2,38 | 49.5 (M:49.5) | Carbamidomethyl: 20; Dimethyl:2H(4): 1, 22 | 94 - 115 | ENSGALP00000010210 | leukocyte cell derived chemotaxin 2 - CGNC Symbol 4754 |
| 2640,3296 | -13,03 | 109.8 (M:109.8) | Carbamidomethyl: 6; Dimethyl:2H(4): 1 | 69 - 93 | ENSGALP00000010210 | leukocyte cell derived chemotaxin 2 - CGNC Symbol 4754 |
| 995,5586 | 1,52 | 46.3 (M:46.3) | Dimethyl:2H(4): 1 | 568 - 575 | ENSGALP00000016536 | heat shock 70kDa protein 4-like - CGNC Symbol 51755 |
| 1564,8111 | 2,11 | 27.3 (M:27.3) | Dimethyl: 1, 6 | 562 - 575 | ENSGALP00000016536 | heat shock 70kDa protein 4-like - CGNC Symbol 51755 |
| 1572,8606 | 1,62 | 47.4 (M:47.4) | Dimethyl:2H(4): 1, 6 | 562 - 575 | ENSGALP00000016536 | heat shock 70kDa protein 4-like - CGNC Symbol 51755 |
| 859,4431 | -11,74 | 20.4 (M:20.4) | Dimethyl:2H(4): 1; Oxidation: 4; Dimethyl: 6 | 675 - 680 | ENSGALP00000016536 | heat shock 70kDa protein 4-like - CGNC Symbol 51755 |
| 880,5382 | -12,36 | 20.5 (M:20.5) | Dimethyl:2H(4): 1, 7 | 62 - 68 | ENSGALP00000016536 | heat shock 70kDa protein 4-like - CGNC Symbol 51755 |
| 1006,5581 | -3,67 | 36.9 (M:36.9) | Dimethyl:2H(4): 1 | 687 - 694 | ENSGALP00000016536 | heat shock 70kDa protein 4-like - CGNC Symbol 51755 |
| 1166,713 | -0,15 | 24.1 (M:24.1) | Dimethyl:2H(4): 1, 1 | 686 - 694 | ENSGALP00000016536 | heat shock 70kDa protein 4-like - CGNC Symbol 51755 |
| 2357,1158 | -1,36 | 47.1 (M:47.1) | Dimethyl:2H(4): 1, 21 | 541 - 561 | ENSGALP00000016536 | heat shock 70kDa protein 4-like - CGNC Symbol 51755 |
| 1159,7413 | 15,39 | 17.8 (M:17.8) | Dimethyl:2H(4): 1; Dimethyl: 10 | 761 - 770 | ENSGALP00000016536 | heat shock 70kDa protein 4-like - CGNC Symbol 51755 |
| 1820,951 | 7,29 | 65.3 (M:65.3) | Dimethyl:2H(4): 1, 16 | 462 - 477 | ENSGALP00000016536 | heat shock 70kDa protein 4-like - CGNC Symbol 51755 |
| 1351,7532 | -3,56 | 29.4 (M:29.4) | Dimethyl: 1, 9 | 186 - 196 | ENSGALP00000016536 | heat shock 70kDa protein 4-like - CGNC Symbol 51755 |
| 1359,814 | 4,26 | 72.0 (M:72.0) | Dimethyl:2H(4): 1, 9 | 186 - 196 | ENSGALP00000016536 | heat shock 70kDa protein 4-like - CGNC Symbol 51755 |
| 1360,6631 | -3,73 | 16.6 (M:16.6) | Dimethyl:2H(4): 1 | 74 - 84 | ENSGALP00000016536 | heat shock 70kDa protein 4-like - CGNC Symbol 51755 |
| 761,4641 | 6,68 | 16.6 (M:16.6) | Oxidation: 5 | 712 - 717 | ENSGALP00000016536 | heat shock 70kDa protein 4-like - CGNC Symbol 51755 |
| 1538,8322 | 1,57 | 81.2 (M:81.2) | Carbamidomethyl: 7; Dimethyl:2H(4): 1 | 304 - 316 | ENSGALP00000016536 | heat shock 70kDa protein 4-like - CGNC Symbol 51755 |
| 1534,8108 | 4,04 | 66.0 (M:66.0) | Carbamidomethyl: 7; Dimethyl: 1 | 304 - 316 | ENSGALP00000016536 | heat shock 70kDa protein 4-like - CGNC Symbol 51755 |
| 1591,8458 | 7,77 | 49.5 (M:49.5) | Carbamidomethyl: 2, 6; Dimethyl: 1, 14 | 375 - 388 | ENSGALP00000016536 | heat shock 70kDa protein 4-like - CGNC Symbol 51755 |
| 1599,8902 | 4,09 | 61.8 (M:61.8) | Carbamidomethyl: 2, 6; Dimethyl:2H(4): 1, 14 | 375 - 388 | ENSGALP00000016536 | heat shock 70kDa protein 4-like - CGNC Symbol 51755 |
| 1421,778 | -0,54 | 31.1 (M:31.1) | Dimethyl: 1 | 222 - 234 | ENSGALP00000016536 | heat shock 70kDa protein 4-like - CGNC Symbol 51755 |
| 1425,8004 | -2,44 | 83.2 (M:83.2) | Dimethyl:2H(4): 1 | 222 - 234 | ENSGALP00000016536 | heat shock 70kDa protein 4-like - CGNC Symbol 51755 |
| 2248,2258 | -6,78 | 28.1 (M:28.1) | Carbamidomethyl: 8; Dimethyl: 2, 16, 18 | 778 - 795 | ENSGALP00000016536 | heat shock 70kDa protein 4-like - CGNC Symbol 51755 |
| 1436,6901 | -6,52 | 37.8 (M:37.8) | Dimethyl:2H(4): 1 | 625 - 635 | ENSGALP00000016536 | heat shock 70kDa protein 4-like - CGNC Symbol 51755 |
| 1403,8 | 7,66 | 45.4 (M:45.4) | Dimethyl: 1 | 582 - 593 | ENSGALP00000016536 | heat shock 70kDa protein 4-like - CGNC Symbol 51755 |
| 1407,83 | 11,05 | 72.5 (M:72.5) | Dimethyl:2H(4): 1 | 582 - 593 | ENSGALP00000016536 | heat shock 70kDa protein 4-like - CGNC Symbol 51755 |
| 1432,6582 | -11,28 | 19.2 (M:19.2) | Dimethyl: 1 | 625 - 635 | ENSGALP00000016536 | heat shock 70kDa protein 4-like - CGNC Symbol 51755 |
| 1597,9815 | -1,4 | 52.8 (M:52.8) | Dimethyl:2H(4): 1, 14 | 111 - 124 | ENSGALP00000016536 | heat shock 70kDa protein 4-like - CGNC Symbol 51755 |
| 1771,9484 | -16,6 | 66.0 (M:66.0) | Dimethyl:2H(4): 1 | 391 - 405 | ENSGALP00000016536 | heat shock 70kDa protein 4-like - CGNC Symbol 51755 |
| 1767,926 | -15,1 | 45.6 (M:45.6) | Dimethyl: 1 | 391 - 405 | ENSGALP00000016536 | heat shock 70kDa protein 4-like - CGNC Symbol 51755 |
| 1908,8488 | -8,53 | 40.2 (M:40.2) | Dimethyl: 1 | 235 - 249 | ENSGALP00000016536 | heat shock 70kDa protein 4-like - CGNC Symbol 51755 |
| 1912,874 | -8,42 | 40.5 (M:40.5) | Dimethyl:2H(4): 1 | 235 - 249 | ENSGALP00000016536 | heat shock 70kDa protein 4-like - CGNC Symbol 51755 |
| 2067,0208 | 2,58 | 47.1 (M:47.1) | Dimethyl: 1 | 438 - 453 | ENSGALP00000028664 | 2'-5'-oligoadenylate synthetase-like - CGNC Symbol 49545 |
| 2071,0443 | 1,81 | 83.7 (M:83.7) | Dimethyl:2H(4): 1 | 438 - 453 | ENSGALP00000028664 | 2'-5'-oligoadenylate synthetase-like - CGNC Symbol 49545 |
| 1915,9931 | 9,69 | 61.7 (M:61.7) | Carbamidomethyl: 6; Dimethyl:2H(4): 1 | 417 - 431 | ENSGALP00000028664 | 2'-5'-oligoadenylate synthetase-like - CGNC Symbol 49545 |
| 1403,7076 | 7,98 | 35.5 (M:35.5) | Dimethyl:2H(4): 1 | 416 - 427 | ENSGALP00000039235 | cold shock domain containing E1 - CGNC Symbol 51115 |
| 1038,5367 | 3,9 | 24.1 (M:24.1) | Dimethyl: 1 | 278 - 286 | ENSGALP00000039235 | cold shock domain containing E1 - CGNC Symbol 51115 |
| 1042,5628 | 4,81 | 34.6 (M:34.6) | Dimethyl:2H(4): 1 | 278 - 286 | ENSGALP00000039235 | cold shock domain containing E1 - CGNC Symbol 51115 |
| 1791,8285 | -2,52 | 74.2 (M:74.2) | Carbamidomethyl: 14; Dimethyl: 1 | 116 - 132 | ENSGALP00000039235 | cold shock domain containing E1 - CGNC Symbol 51115 |
| 1795,8545 | -2,04 | 56.9 (M:56.9) | Carbamidomethyl: 14; Dimethyl:2H(4): 1 | 116 - 132 | ENSGALP00000039235 | cold shock domain containing E1 - CGNC Symbol 51115 |
| 1279,7651 | 2,43 | 35.0 (M:35.0) | Dimethyl: 1, 2 | 90 - 99 | ENSGALP00000039235 | cold shock domain containing E1 - CGNC Symbol 51115 |
| 1287,8165 | 3,3 | 55.8 (M:55.8) | Dimethyl:2H(4): 1, 2 | 90 - 99 | ENSGALP00000039235 | cold shock domain containing E1 - CGNC Symbol 51115 |
| 1601,7953 | -2,44 | 18.0 (M:18.0) | Carbamidomethyl: 10; Dimethyl: 1 | 33 - 45 | ENSGALP00000039235 | cold shock domain containing E1 - CGNC Symbol 51115 |
| 1605,8182 | -3,79 | 35.6 (M:35.6) | Carbamidomethyl: 10; Dimethyl:2H(4): 1 | 33 - 45 | ENSGALP00000039235 | cold shock domain containing E1 - CGNC Symbol 51115 |
| 845,5172 | 4,78 | 18.6 (M:18.6) | Dimethyl: 7 | 289 - 295 | ENSGALP00000039235 | cold shock domain containing E1 - CGNC Symbol 51115 |
| 996,5073 | -7,64 | 28.7 (M:28.7) | Dimethyl: 1 | 195 - 202 | ENSGALP00000039235 | cold shock domain containing E1 - CGNC Symbol 51115 |
| 1000,5373 | -2,75 | 29.2 (M:29.2) | Dimethyl:2H(4): 1 | 195 - 202 | ENSGALP00000039235 | cold shock domain containing E1 - CGNC Symbol 51115 |
| 1432,817 | 8,65 | 17.5 (M:17.5) | Dimethyl: 7, 13 | 428 - 440 | ENSGALP00000039235 | cold shock domain containing E1 - CGNC Symbol 51115 |
| 1091,636 | 24,22 | 25.9 (M:25.9) | Dimethyl: 9 | 158 - 166 | ENSGALP00000039235 | cold shock domain containing E1 - CGNC Symbol 51115 |
| 742,4669 | 18,57 | 17.3 (M:17.3) | Dimethyl: 1, 6 | 1,006 - 1,011 | ENSGALP00000013029 | splicing factor 3b subunit 1 - CGNC Symbol 6082 |
| 1495,7083 | 10,58 | 58.7 (M:58.7) | Dimethyl: 1 | 299 - 312 | ENSGALP00000013029 | splicing factor 3b subunit 1 - CGNC Symbol 6082 |
| 1859,9455 | 3 | 56.0 (M:56.0) | Dimethyl: 1 | 251 - 268 | ENSGALP00000013029 | splicing factor 3b subunit 1 - CGNC Symbol 6082 |
| 1418,8488 | -8,29 | 23.9 (M:23.9) | Dimethyl:2H(4): 1, 11 | 560 - 570 | ENSGALP00000013029 | splicing factor 3b subunit 1 - CGNC Symbol 6082 |
| 837,4347 | -0,61 | 31.1 (M:31.1) | Dimethyl: 1, 6 | 1,284 - 1,289 | ENSGALP00000013029 | splicing factor 3b subunit 1 - CGNC Symbol 6082 |
| 1336,7972 | -3,68 | 17.2 (M:17.2) | Dimethyl: 1, 6 | 1,006 - 1,016 | ENSGALP00000013029 | splicing factor 3b subunit 1 - CGNC Symbol 6082 |
| 1306,7863 | -1,42 | 40.4 (M:40.4) | Dimethyl: 1 | 411 - 422 | ENSGALP00000013029 | splicing factor 3b subunit 1 - CGNC Symbol 6082 |
| 1310,8021 | -8,51 | 38.1 (M:38.1) | Dimethyl:2H(4): 1 | 411 - 422 | ENSGALP00000013029 | splicing factor 3b subunit 1 - CGNC Symbol 6082 |
| 1757,8891 | -4,07 | 19.1 (M:19.1) | Carbamidomethyl: 5 | 1,028 - 1,042 | ENSGALP00000013029 | splicing factor 3b subunit 1 - CGNC Symbol 6082 |
| 2265,1483 | 9,82 | 58.6 (M:58.6) | Dimethyl: 1 | 746 - 764 | ENSGALP00000013029 | splicing factor 3b subunit 1 - CGNC Symbol 6082 |
| 1761,9067 | 7,91 | 54.0 (M:54.0) | Carbamidomethyl: 5; Dimethyl: 1 | 1,243 - 1,256 | ENSGALP00000013029 | splicing factor 3b subunit 1 - CGNC Symbol 6082 |
| 975,5128 | -5,31 | 24.4 (M:24.4) | Dimethyl: 1, 8 | 43313 | ENSGALP00000042479 | Hsap of 1: stress induced phosphoprotein 1 - HGNC:11387 |
| 1969,012 | -0,91 | 85.5 (M:85.5) | Dimethyl: 1, 8 | 93 - 109 | ENSGALP00000042479 | Hsap of 1: stress induced phosphoprotein 1 - HGNC:11387 |
| 1977,0637 | -0,16 | 77.9 (M:77.9) | Dimethyl:2H(4): 1, 8 | 93 - 109 | ENSGALP00000042479 | Hsap of 1: stress induced phosphoprotein 1 - HGNC:11387 |
| 1411,7605 | -3,32 | 36.2 (M:36.2) | Dimethyl: 1 | 45962 | ENSGALP00000042479 | Hsap of 1: stress induced phosphoprotein 1 - HGNC:11387 |
| 1415,7847 | -3,99 | 28.7 (M:28.7) | Dimethyl:2H(4): 1 | 45962 | ENSGALP00000042479 | Hsap of 1: stress induced phosphoprotein 1 - HGNC:11387 |
| 2198,1565 | 7,34 | 71.2 (M:71.2) | Dimethyl:2H(4): 1 | 26 - 44 | ENSGALP00000042479 | Hsap of 1: stress induced phosphoprotein 1 - HGNC:11387 |
| 2194,1172 | 0,9 | 58.8 (M:58.8) | Dimethyl: 1 | 26 - 44 | ENSGALP00000042479 | Hsap of 1: stress induced phosphoprotein 1 - HGNC:11387 |
| 1585,8663 | -11,53 | 15.0 (M:15.0) | Dimethyl:2H(4): 1 | 161 - 174 | ENSGALP00000042479 | Hsap of 1: stress induced phosphoprotein 1 - HGNC:11387 |
| 1188,7101 | 0,17 | 18.3 (M:18.3) | Dimethyl: 1, 1 | 78 - 87 | ENSGALP00000042479 | Hsap of 1: stress induced phosphoprotein 1 - HGNC:11387 |
| 1036,603 | -5,54 | 32.4 (M:32.4) | Dimethyl:2H(4): 1 | 79 - 87 | ENSGALP00000042479 | Hsap of 1: stress induced phosphoprotein 1 - HGNC:11387 |
| 1327,6612 | 2,52 | 31.8 (M:31.8) | Dimethyl:2H(4): 1 | 241 - 251 | ENSGALP00000042479 | Hsap of 1: stress induced phosphoprotein 1 - HGNC:11387 |
| 928,5299 | -17,54 | 35.7 (M:35.7) | Dimethyl: 1 | 154 - 160 | ENSGALP00000042479 | Hsap of 1: stress induced phosphoprotein 1 - HGNC:11387 |
| 932,5588 | -13,46 | 39.4 (M:39.4) | Dimethyl:2H(4): 1 | 154 - 160 | ENSGALP00000042479 | Hsap of 1: stress induced phosphoprotein 1 - HGNC:11387 |
| 1032,593 | 9,04 | 19.7 (M:19.7) | Dimethyl: 1 | 79 - 87 | ENSGALP00000042479 | Hsap of 1: stress induced phosphoprotein 1 - HGNC:11387 |
| 1613,9199 | 2,47 | 17.7 (M:17.7) | Dimethyl:2H(4): 9; Dimethyl: 1 | 161 - 174 | ENSGALP00000042479 | Hsap of 1: stress induced phosphoprotein 1 - HGNC:11387 |
| 1664,9079 | 4,36 | 16.1 (M:16.1) | Dimethyl: 1, 14 | 79 - 92 | ENSGALP00000042479 | Hsap of 1: stress induced phosphoprotein 1 - HGNC:11387 |
| 1531,8859 | -1,57 | 47.1 (M:47.1) | Dimethyl: 1, 13 | 124 - 136 | ENSGALP00000042479 | Hsap of 1: stress induced phosphoprotein 1 - HGNC:11387 |
| 1280,7055 | 18,64 | 16.8 (M:16.8) | Dimethyl:2H(4): 1; Oxidation: 1; Dimethyl: 8 | 43374 | ENSGALP00000042479 | Hsap of 1: stress induced phosphoprotein 1 - HGNC:11387 |
| 1472,7794 | 18,2 | 56.6 (M:56.6) | Carbamidomethyl: 4; Dimethyl: 1 | 130 - 141 | ENSGALP00000011961 | phosphatidylethanolamine binding protein 1 - CGNC Symbol 5589 |
| 1476,8024 | 16,72 | 55.0 (M:55.0) | Carbamidomethyl: 4; Dimethyl:2H(4): 1 | 130 - 141 | ENSGALP00000011961 | phosphatidylethanolamine binding protein 1 - CGNC Symbol 5589 |
| 2748,4072 | 10,85 | 29.5 (M:29.5) | Carbamidomethyl: 19; Dimethyl: 1, 23 | 40 - 62 | ENSGALP00000011961 | phosphatidylethanolamine binding protein 1 - CGNC Symbol 5589 |
| 1800,9323 | -2,43 | 36.9 (M:36.9) | Dimethyl: 1, 13 | 81 - 93 | ENSGALP00000011961 | phosphatidylethanolamine binding protein 1 - CGNC Symbol 5589 |
| 1380,7563 | 0,06 | 52.8 (M:52.8) | Dimethyl: 1, 10 | 120 - 129 | ENSGALP00000011961 | phosphatidylethanolamine binding protein 1 - CGNC Symbol 5589 |
| 1588,856 | -1,35 | 87.3 (M:87.3) | Dimethyl: 1 | 63 - 76 | ENSGALP00000011961 | phosphatidylethanolamine binding protein 1 - CGNC Symbol 5589 |
| 1592,8806 | -1,64 | 79.0 (M:79.0) | Dimethyl:2H(4): 1 | 63 - 76 | ENSGALP00000011961 | phosphatidylethanolamine binding protein 1 - CGNC Symbol 5589 |
| 2795,4735 | -12,71 | 39.8 (M:39.8) | Dimethyl: 19 | 46054 | ENSGALP00000011961 | phosphatidylethanolamine binding protein 1 - CGNC Symbol 5589 |
| 2837,5194 | -0,09 | 60.0 (M:60.0) | Acetyl: 1; Dimethyl: 19 | 46054 | ENSGALP00000011961 | phosphatidylethanolamine binding protein 1 - CGNC Symbol 5589 |
| 2823,5019 | -13,63 | 79.1 (M:79.1) | Dimethyl: 1, 19 | 46054 | ENSGALP00000011961 | phosphatidylethanolamine binding protein 1 - CGNC Symbol 5589 |
| 2841,4869 | -20,34 | 32.1 (M:32.1) | Dimethyl:2H(4): 19; Acetyl: 1 | 46054 | ENSGALP00000011961 | phosphatidylethanolamine binding protein 1 - CGNC Symbol 5589 |
| 833,4631 | 5,13 | 15.1 (M:15.1) | Dimethyl: 1 | 43 - 49 | ENSGALP00000016363 | heterochromatin protein 1 binding protein 3 - CGNC Symbol 7661 |
| 1103,6033 | 23,69 | 51.5 (M:51.5) | Dimethyl: 1, 8 | 285 - 292 | ENSGALP00000016363 | heterochromatin protein 1 binding protein 3 - CGNC Symbol 7661 |
| 1111,6541 | 24,06 | 19.3 (M:19.3) | Dimethyl:2H(4): 1, 8 | 285 - 292 | ENSGALP00000016363 | heterochromatin protein 1 binding protein 3 - CGNC Symbol 7661 |
| 837,4659 | -21,54 | 20.8 (M:20.8) | Dimethyl:2H(4): 1 | 43 - 49 | ENSGALP00000016363 | heterochromatin protein 1 binding protein 3 - CGNC Symbol 7661 |
| 1001,5513 | -11,31 | 18.8 (M:18.8) | Dimethyl: 9 | 313 - 321 | ENSGALP00000016363 | heterochromatin protein 1 binding protein 3 - CGNC Symbol 7661 |
| 1716,9587 | -5,6 | 39.4 (M:39.4) | Dimethyl: 1, 5 | 187 - 199 | ENSGALP00000016363 | heterochromatin protein 1 binding protein 3 - CGNC Symbol 7661 |
| 1034,5489 | -2,68 | 34.5 (M:34.5) | Dimethyl: 1 | 192 - 199 | ENSGALP00000016363 | heterochromatin protein 1 binding protein 3 - CGNC Symbol 7661 |
| 1137,7249 | -9,27 | 15.7 (M:15.7) | Dimethyl: 1, 9 | 295 - 303 | ENSGALP00000016363 | heterochromatin protein 1 binding protein 3 - CGNC Symbol 7661 |
| 1056,6076 | -8 | 15.9 (M:15.9) | Dimethyl: 9 | 304 - 312 | ENSGALP00000016363 | heterochromatin protein 1 binding protein 3 - CGNC Symbol 7661 |
| 1038,5764 | -0,35 | 30.8 (M:30.8) | Dimethyl:2H(4): 1 | 192 - 199 | ENSGALP00000016363 | heterochromatin protein 1 binding protein 3 - CGNC Symbol 7661 |
| 2436,3048 | -4,04 | 34.6 (M:34.6) | Dimethyl: 1, 19 | 368 - 387 | ENSGALP00000016363 | heterochromatin protein 1 binding protein 3 - CGNC Symbol 7661 |
| 1677,0049 | 6,18 | 104.1 (M:104.1) | Dimethyl: 1, 15 | 15 - 29 | ENSGALP00000016363 | heterochromatin protein 1 binding protein 3 - CGNC Symbol 7661 |
| 938,5104 | -9,46 | 18.4 (M:18.4) | Dimethyl: 8 | 276 - 283 | ENSGALP00000016363 | heterochromatin protein 1 binding protein 3 - CGNC Symbol 7661 |
| 1613,9199 | 11,73 | 79.3 (M:79.3) | Dimethyl: 1 | 133 - 147 | ENSGALP00000016363 | heterochromatin protein 1 binding protein 3 - CGNC Symbol 7661 |
| 1315,7466 | -11,71 | 93.4 (M:93.4) | Dimethyl: 1 | 260 - 270 | ENSGALP00000016363 | heterochromatin protein 1 binding protein 3 - CGNC Symbol 7661 |
| 1319,7705 | -12,61 | 72.3 (M:72.3) | Dimethyl:2H(4): 1 | 260 - 270 | ENSGALP00000016363 | heterochromatin protein 1 binding protein 3 - CGNC Symbol 7661 |
| 1855,8387 | 4,44 | 48.3 (M:48.3) | Dimethyl: 1, 16 | 128 - 143 | ENSGALP00000015128 | lamin B receptor - CGNC Symbol 49736 |
| 1172,6394 | 4,31 | 31.2 (M:31.2) | Dimethyl: 1, 2 | 150 - 158 | ENSGALP00000015128 | lamin B receptor - CGNC Symbol 49736 |
| 2051,1204 | 3,55 | 116.1 (M:116.1) | Dimethyl: 1, 11 | 109 - 126 | ENSGALP00000015128 | lamin B receptor - CGNC Symbol 49736 |
| 2059,1574 | -2,9 | 44.6 (M:44.6) | Dimethyl:2H(4): 1, 11 | 109 - 126 | ENSGALP00000015128 | lamin B receptor - CGNC Symbol 49736 |
| 2207,2322 | 8,14 | 15.9 (M:15.9) | Dimethyl: 1, 11 | 109 - 127 | ENSGALP00000015128 | lamin B receptor - CGNC Symbol 49736 |
| 876,5423 | -14,89 | 16.2 (M:16.2) | Dimethyl: 7 | 182 - 188 | ENSGALP00000015128 | lamin B receptor - CGNC Symbol 49736 |
| 1021,5763 | 8,49 | 48.5 (M:48.5) | Dimethyl: 1 | 159 - 166 | ENSGALP00000015128 | lamin B receptor - CGNC Symbol 49736 |
| 1168,6695 | 0,94 | 64.6 (M:64.6) | Dimethyl: 1 | 272 - 282 | ENSGALP00000015128 | lamin B receptor - CGNC Symbol 49736 |
| 1124,5271 | -11,9 | 47.9 (M:47.9) | Dimethyl: 1 | 42156 | ENSGALP00000015128 | lamin B receptor - CGNC Symbol 49736 |
| 1172,6872 | -5,45 | 50.8 (M:50.8) | Dimethyl:2H(4): 1 | 272 - 282 | ENSGALP00000015128 | lamin B receptor - CGNC Symbol 49736 |
| 835,4311 | 0,32 | 37.4 (M:37.4) | Dimethyl: 1 | 199 - 205 | ENSGALP00000015128 | lamin B receptor - CGNC Symbol 49736 |
| 839,4535 | -2,91 | 23.1 (M:23.1) | Dimethyl:2H(4): 1 | 199 - 205 | ENSGALP00000015128 | lamin B receptor - CGNC Symbol 49736 |
| 922,4803 | 2,39 | 42.2 (M:42.2) | Dimethyl: 1 | 588 - 594 | ENSGALP00000015128 | lamin B receptor - CGNC Symbol 49736 |
| 835,4762 | -19,42 | 15.1 (M:15.1) | Dimethyl: 1, 7 | 371 - 377 | ENSGALP00000015128 | lamin B receptor - CGNC Symbol 49736 |
| 1658,951 | -13,12 | 15.4 (M:15.4) | Dimethyl: 1, 6, 14 | 183 - 196 | ENSGALP00000015128 | lamin B receptor - CGNC Symbol 49736 |
| 1424,6612 | -10,38 | 19.5 (M:19.5) | Oxidation: 7; Dimethyl: 9 | 224 - 234 | ENSGALP00000010358 | p21 protein (Cdc42/Rac)-activated kinase 2 - CGNC Symbol 4842 |
| 1790,8738 | -10,11 | 53.8 (M:53.8) | Acetyl: 1; Dimethyl: 9 | 42767 | ENSGALP00000010358 | p21 protein (Cdc42/Rac)-activated kinase 2 - CGNC Symbol 4842 |
| 2880,529 | 12,72 | 38.6 (M:38.6) | Dimethyl: 1, 7, 17 | 18 - 43 | ENSGALP00000010358 | p21 protein (Cdc42/Rac)-activated kinase 2 - CGNC Symbol 4842 |
| 1180,6839 | -21,12 | 63.6 (M:63.6) | Dimethyl: 1, 9 | 490 - 498 | ENSGALP00000010358 | p21 protein (Cdc42/Rac)-activated kinase 2 - CGNC Symbol 4842 |
| 985,578 | -15,08 | 16.8 (M:16.8) | Dimethyl: 1, 9 | 235 - 243 | ENSGALP00000010358 | p21 protein (Cdc42/Rac)-activated kinase 2 - CGNC Symbol 4842 |
| 989,6034 | -14,72 | 17.9 (M:17.9) | Dimethyl:2H(4): 1; Dimethyl: 9 | 235 - 243 | ENSGALP00000010358 | p21 protein (Cdc42/Rac)-activated kinase 2 - CGNC Symbol 4842 |
| 1188,7558 | -2,76 | 34.5 (M:34.5) | Dimethyl:2H(4): 1, 9 | 490 - 498 | ENSGALP00000010358 | p21 protein (Cdc42/Rac)-activated kinase 2 - CGNC Symbol 4842 |
| 2106,1018 | 5 | 98.1 (M:98.1) | Carbamidomethyl: 2; Dimethyl: 1 | 348 - 364 | ENSGALP00000010358 | p21 protein (Cdc42/Rac)-activated kinase 2 - CGNC Symbol 4842 |
| 2110,1281 | 5,53 | 59.2 (M:59.2) | Carbamidomethyl: 2; Dimethyl:2H(4): 1 | 348 - 364 | ENSGALP00000010358 | p21 protein (Cdc42/Rac)-activated kinase 2 - CGNC Symbol 4842 |
| 1952,9325 | -14,53 | 16.4 (M:16.4) | Dimethyl: 1 | 398 - 414 | ENSGALP00000010358 | p21 protein (Cdc42/Rac)-activated kinase 2 - CGNC Symbol 4842 |
| 1409,7502 | 0,43 | 30.1 (M:30.1) | Dimethyl: 1 | 23 - 35 | ENSGALP00000033650 | FYN binding protein - CGNC Symbol 2773 |
| 1441,7931 | 9,2 | 15.1 (M:15.1) | Dimethyl: 1, 8 | 544 - 555 | ENSGALP00000033650 | FYN binding protein - CGNC Symbol 2773 |
| 2057,1845 | 16,67 | 23.7 (M:23.7) | Dimethyl: 1, 7 | 349 - 366 | ENSGALP00000033650 | FYN binding protein - CGNC Symbol 2773 |
| 798,4511 | 0,33 | 16.4 (M:16.4) | Dimethyl: 1 | 807 - 812 | ENSGALP00000033650 | FYN binding protein - CGNC Symbol 2773 |
| 1161,6384 | 0,75 | 23.4 (M:23.4) | Dimethyl: 1 | 93 - 102 | ENSGALP00000033650 | FYN binding protein - CGNC Symbol 2773 |
| 3385,744 | -1 | 72.6 (M:72.6) | Dimethyl: 1 | 383 - 417 | ENSGALP00000033650 | FYN binding protein - CGNC Symbol 2773 |
| 1360,8239 | 13,18 | 44.9 (M:44.9) | Dimethyl: 1 | 508 - 519 | ENSGALP00000033650 | FYN binding protein - CGNC Symbol 2773 |
| 1099,5981 | -11,36 | 21.2 (M:21.2) | Dimethyl: 1 | 535 - 543 | ENSGALP00000033650 | FYN binding protein - CGNC Symbol 2773 |
| 1185,6731 | 2,58 | 24.2 (M:24.2) | Carbamidomethyl: 4; Dimethyl: 1, 2, 9 | 556 - 564 | ENSGALP00000033650 | FYN binding protein - CGNC Symbol 2773 |
| 1189,6976 | 2,08 | 21.8 (M:21.8) | Carbamidomethyl: 4; Dimethyl:2H(4): 1; Dimethyl: 2, 9 | 556 - 564 | ENSGALP00000033650 | FYN binding protein - CGNC Symbol 2773 |
| 2766,1841 | -17,91 | 107.6 (M:107.6) | Dimethyl: 1 | 585 - 609 | ENSGALP00000033650 | FYN binding protein - CGNC Symbol 2773 |
| 857,53 | -22,71 | 20.9 (M:20.9) | Dimethyl: 1, 7 | 342 - 348 | ENSGALP00000033650 | FYN binding protein - CGNC Symbol 2773 |
| 1783,9507 | -7,24 | 17.2 (M:17.2) | Oxidation: 9; Dimethyl: 13, 16 | 223 - 238 | ENSGALP00000033650 | FYN binding protein - CGNC Symbol 2773 |
| 1917,0951 | -23,83 | 17.9 (M:17.9) | Dimethyl:2H(4): 6, 11 | 77 - 92 | ENSGALP00000033650 | FYN binding protein - CGNC Symbol 2773 |
| 1118,6327 | 20,98 | 15.7 (M:15.7) | Dimethyl: 9 | 123 - 131 | ENSGALP00000033650 | FYN binding protein - CGNC Symbol 2773 |
| 1015,5922 | -22,04 | 15.3 (M:15.3) | Dimethyl: 1, 1, 10 | 28 - 37 | ENSGALP00000003584 | H3 histone family 3B - CGNC Symbol 49711 |
| 747,4665 | 0,55 | 32.1 (M:32.1) | Dimethyl:2H(4): 1 | 124 - 129 | ENSGALP00000003584 | H3 histone family 3B - CGNC Symbol 49711 |
| 744,4755 | 8,97 | 31.8 (M:31.8) | Dimethyl: 1, 6 | 118 - 123 | ENSGALP00000003584 | H3 histone family 3B - CGNC Symbol 49711 |
| 760,4681 | 5,77 | 15.8 (M:15.8) | Oxidation: 4; Dimethyl: 1, 6 | 118 - 123 | ENSGALP00000003584 | H3 histone family 3B - CGNC Symbol 49711 |
| 906,4794 | -15,1 | 48.2 (M:48.2) | Dimethyl: 1, 7 | 74 - 80 | ENSGALP00000003584 | H3 histone family 3B - CGNC Symbol 49711 |
| 914,5293 | -15,33 | 43.0 (M:43.0) | Dimethyl:2H(4): 1, 7 | 74 - 80 | ENSGALP00000003584 | H3 histone family 3B - CGNC Symbol 49711 |
| 863,549 | -0,99 | 44.3 (M:44.3) | Dimethyl:2H(4): 1 | 58 - 64 | ENSGALP00000003584 | H3 histone family 3B - CGNC Symbol 49711 |
| 1060,6134 | -12,02 | 28.8 (M:28.8) | Dimethyl: 1 | 42 - 50 | ENSGALP00000003584 | H3 histone family 3B - CGNC Symbol 49711 |
| 1064,6393 | -11,28 | 29.9 (M:29.9) | Dimethyl:2H(4): 1 | 42 - 50 | ENSGALP00000003584 | H3 histone family 3B - CGNC Symbol 49711 |
| 859,5209 | -4,51 | 56.6 (M:56.6) | Dimethyl: 1 | 58 - 64 | ENSGALP00000003584 | H3 histone family 3B - CGNC Symbol 49711 |
| 1391,7602 | 5,22 | 75.8 (M:75.8) | Dimethyl: 1, 7 | 74 - 84 | ENSGALP00000003584 | H3 histone family 3B - CGNC Symbol 49711 |
| 1395,7908 | 9,17 | 51.0 (M:51.0) | Dimethyl:2H(4): 1; Dimethyl: 7 | 74 - 84 | ENSGALP00000003584 | H3 histone family 3B - CGNC Symbol 49711 |
| 900,5706 | 0,7 | 20.1 (M:20.1) | Dimethyl: 1, 7 | 117 - 123 | ENSGALP00000003584 | H3 histone family 3B - CGNC Symbol 49711 |
| 743,433 | -10,78 | 43.3 (M:43.3) | Dimethyl: 1 | 124 - 129 | ENSGALP00000003584 | H3 histone family 3B - CGNC Symbol 49711 |
| 1565,8 | -6,69 | 74.9 (M:74.9) | Dimethyl: 1, 7 | 294 - 306 | ENSGALP00000039872 | regulator of chromosome condensation 2 - CGNC Symbol 287 |
| 916,4827 | -6,55 | 38.6 (M:38.6) | Dimethyl: 1 | 446 - 452 | ENSGALP00000039872 | regulator of chromosome condensation 2 - CGNC Symbol 287 |
| 920,5043 | -10,37 | 31.5 (M:31.5) | Dimethyl:2H(4): 1 | 446 - 452 | ENSGALP00000039872 | regulator of chromosome condensation 2 - CGNC Symbol 287 |
| 1940,0387 | 19,01 | 64.3 (M:64.3) | Carbamidomethyl: 4; Dimethyl: 1, 16 | 266 - 282 | ENSGALP00000039872 | regulator of chromosome condensation 2 - CGNC Symbol 287 |
| 1166,5914 | -6,22 | 48.3 (M:48.3) | Carbamidomethyl: 3; Dimethyl: 1 | 74 - 83 | ENSGALP00000039872 | regulator of chromosome condensation 2 - CGNC Symbol 287 |
| 1319,6967 | -0,03 | 26.3 (M:26.3) | Dimethyl: 1 | 63 - 73 | ENSGALP00000039872 | regulator of chromosome condensation 2 - CGNC Symbol 287 |
| 1691,0339 | 7,36 | 88.1 (M:88.1) | Dimethyl: 1, 2 | 251 - 265 | ENSGALP00000039872 | regulator of chromosome condensation 2 - CGNC Symbol 287 |
| 1321,6251 | -15,56 | 63.0 (M:63.0) | Carbamidomethyl: 5; Dimethyl: 1 | 233 - 242 | ENSGALP00000039872 | regulator of chromosome condensation 2 - CGNC Symbol 287 |
| 879,4606 | -13,37 | 28.5 (M:28.5) | Dimethyl: 1 | 310 - 316 | ENSGALP00000039872 | regulator of chromosome condensation 2 - CGNC Symbol 287 |
| 1325,6523 | -13,94 | 52.0 (M:52.0) | Carbamidomethyl: 5; Dimethyl:2H(4): 1 | 233 - 242 | ENSGALP00000039872 | regulator of chromosome condensation 2 - CGNC Symbol 287 |
| 2356,0614 | -2,05 | 43.7 (M:43.7) | Carbamidomethyl: 8; Dimethyl: 1, 21 | 205 - 225 | ENSGALP00000039872 | regulator of chromosome condensation 2 - CGNC Symbol 287 |
| 832,43 | -19,71 | 23.4 (M:23.4) | Dimethyl: 1 | 103 - 108 | ENSGALP00000039872 | regulator of chromosome condensation 2 - CGNC Symbol 287 |
| 2108,0575 | -3,99 | 93.4 (M:93.4) | Dimethyl: 1, 19 | 144 - 162 | ENSGALP00000039872 | regulator of chromosome condensation 2 - CGNC Symbol 287 |
| 1799,0062 | -1,85 | 48.6 (M:48.6) | Carbamidomethyl: 15; Dimethyl: 1 | 127 - 143 | ENSGALP00000039872 | regulator of chromosome condensation 2 - CGNC Symbol 287 |
| 1433,8442 | -2,3 | 17.4 (M:17.4) | Dimethyl: 1 | 253 - 265 | ENSGALP00000039872 | regulator of chromosome condensation 2 - CGNC Symbol 287 |
| 1260,6077 | -6,6 | 68.3 (M:68.3) | Dimethyl: 1 | 283 - 293 | ENSGALP00000039872 | regulator of chromosome condensation 2 - CGNC Symbol 287 |
| 1108,7057 | -19,97 | 16.6 (M:16.6) | Dimethyl:2H(4): 1; Dimethyl: 7 | 244 - 252 | ENSGALP00000039872 | regulator of chromosome condensation 2 - CGNC Symbol 287 |
| 2436,2854 | 10,76 | 90.2 (M:90.2) | Dimethyl: 1 | 412 - 433 | ENSGALP00000039872 | regulator of chromosome condensation 2 - CGNC Symbol 287 |
| 2440,3063 | 9,04 | 85.9 (M:85.9) | Dimethyl:2H(4): 1 | 412 - 433 | ENSGALP00000039872 | regulator of chromosome condensation 2 - CGNC Symbol 287 |
| 1906,0964 | 21,5 | 16.0 (M:16.0) | Carbamidomethyl: 13; Dimethyl:2H(4): 1, 3; Dimethyl: 17 | 47088 | ENSGALP00000039872 | regulator of chromosome condensation 2 - CGNC Symbol 287 |
| 1346,7117 | 4 | 70.9 (M:70.9) | Dimethyl: 1, 10 | 69 - 79 | ENSGALP00000041526 | Gallus gallus histone cluster 1 H4-VI germinal H4 (similar to human histone  cluster 1 class H4 genes) (HIST1H46) mRNA. - RefSeq mRNA NM_001037845 |
| 1198,6643 | 7,4 | 63.7 (M:63.7) | Dimethyl:2H(4): 1, 10 | 69 - 78 | ENSGALP00000041526 | Gallus gallus histone cluster 1 H4-VI germinal H4 (similar to human histone  cluster 1 class H4 genes) (HIST1H46) mRNA. - RefSeq mRNA NM_001037845 |
| 1190,6092 | 3,35 | 81.1 (M:81.1) | Dimethyl: 1, 10 | 69 - 78 | ENSGALP00000041526 | Gallus gallus histone cluster 1 H4-VI germinal H4 (similar to human histone  cluster 1 class H4 genes) (HIST1H46) mRNA. - RefSeq mRNA NM_001037845 |
| 1194,6209 | -7,83 | 54.0 (M:54.0) | Dimethyl:2H(4): 1; Dimethyl: 10 | 69 - 78 | ENSGALP00000041526 | Gallus gallus histone cluster 1 H4-VI germinal H4 (similar to human histone  cluster 1 class H4 genes) (HIST1H46) mRNA. - RefSeq mRNA NM_001037845 |
| 1353,8001 | 11,28 | 17.8 (M:17.8) | Dimethyl: 8 | 25 - 36 | ENSGALP00000041526 | Gallus gallus histone cluster 1 H4-VI germinal H4 (similar to human histone  cluster 1 class H4 genes) (HIST1H46) mRNA. - RefSeq mRNA NM_001037845 |
| 1353,7932 | 6,15 | 43.5 (M:43.5) | Dimethyl: 1 | 25 - 36 | ENSGALP00000041526 | Gallus gallus histone cluster 1 H4-VI germinal H4 (similar to human histone  cluster 1 class H4 genes) (HIST1H46) mRNA. - RefSeq mRNA NM_001037845 |
| 1389,8961 | 21,39 | 39.0 (M:39.0) | Dimethyl:2H(4): 1, 8 | 25 - 36 | ENSGALP00000041526 | Gallus gallus histone cluster 1 H4-VI germinal H4 (similar to human histone  cluster 1 class H4 genes) (HIST1H46) mRNA. - RefSeq mRNA NM_001037845 |
| 1385,8417 | 0,34 | 30.6 (M:30.6) | Dimethyl:2H(4): 8; Dimethyl: 1 | 25 - 36 | ENSGALP00000041526 | Gallus gallus histone cluster 1 H4-VI germinal H4 (similar to human histone  cluster 1 class H4 genes) (HIST1H46) mRNA. - RefSeq mRNA NM_001037845 |
| 1364,7483 | -3,59 | 68.2 (M:68.2) | Dimethyl: 1 | 46 - 56 | ENSGALP00000041526 | Gallus gallus histone cluster 1 H4-VI germinal H4 (similar to human histone  cluster 1 class H4 genes) (HIST1H46) mRNA. - RefSeq mRNA NM_001037845 |
| 1368,7739 | -3,24 | 29.6 (M:29.6) | Dimethyl:2H(4): 1 | 46 - 56 | ENSGALP00000041526 | Gallus gallus histone cluster 1 H4-VI germinal H4 (similar to human histone  cluster 1 class H4 genes) (HIST1H46) mRNA. - RefSeq mRNA NM_001037845 |
| 1381,8041 | -8,76 | 83.7 (M:83.7) | Dimethyl: 1, 8 | 25 - 36 | ENSGALP00000041526 | Gallus gallus histone cluster 1 H4-VI germinal H4 (similar to human histone  cluster 1 class H4 genes) (HIST1H46) mRNA. - RefSeq mRNA NM_001037845 |
| 1208,647 | -4,24 | 80.0 (M:80.0) | Dimethyl: 1 | 47 - 56 | ENSGALP00000041526 | Gallus gallus histone cluster 1 H4-VI germinal H4 (similar to human histone  cluster 1 class H4 genes) (HIST1H46) mRNA. - RefSeq mRNA NM_001037845 |
| 1021,6325 | -1,76 | 33.6 (M:33.6) | Dimethyl:2H(4): 1 | 61 - 68 | ENSGALP00000041526 | Gallus gallus histone cluster 1 H4-VI germinal H4 (similar to human histone  cluster 1 class H4 genes) (HIST1H46) mRNA. - RefSeq mRNA NM_001037845 |
| 1385,83 | -8,17 | 28.0 (M:28.0) | Dimethyl:2H(4): 1; Dimethyl: 8 | 25 - 36 | ENSGALP00000041526 | Gallus gallus histone cluster 1 H4-VI germinal H4 (similar to human histone  cluster 1 class H4 genes) (HIST1H46) mRNA. - RefSeq mRNA NM_001037845 |
| 1494,8543 | 13 | 20.5 (M:20.5) | Dimethyl: 1 | 81 - 93 | ENSGALP00000041526 | Gallus gallus histone cluster 1 H4-VI germinal H4 (similar to human histone  cluster 1 class H4 genes) (HIST1H46) mRNA. - RefSeq mRNA NM_001037845 |
| 1522,8774 | 7,41 | 111.4 (M:111.4) | Dimethyl: 1, 12 | 81 - 93 | ENSGALP00000041526 | Gallus gallus histone cluster 1 H4-VI germinal H4 (similar to human histone  cluster 1 class H4 genes) (HIST1H46) mRNA. - RefSeq mRNA NM_001037845 |
| 1538,8721 | 7,16 | 66.3 (M:66.3) | Oxidation: 5; Dimethyl: 1, 12 | 81 - 93 | ENSGALP00000041526 | Gallus gallus histone cluster 1 H4-VI germinal H4 (similar to human histone  cluster 1 class H4 genes) (HIST1H46) mRNA. - RefSeq mRNA NM_001037845 |
| 1550,9582 | -2,13 | 33.2 (M:33.2) | Dimethyl:2H(4): 1, 1, 13; Oxidation: 6 | 80 - 92 | ENSGALP00000041526 | Gallus gallus histone cluster 1 H4-VI germinal H4 (similar to human histone  cluster 1 class H4 genes) (HIST1H46) mRNA. - RefSeq mRNA NM_001037845 |
| 742,3754 | -2,24 | 49.3 (M:49.3) | Dimethyl: 1 | 97 - 103 | ENSGALP00000041526 | Gallus gallus histone cluster 1 H4-VI germinal H4 (similar to human histone  cluster 1 class H4 genes) (HIST1H46) mRNA. - RefSeq mRNA NM_001037845 |
| 746,4009 | -1,71 | 26.0 (M:26.0) | Dimethyl:2H(4): 1 | 97 - 103 | ENSGALP00000041526 | Gallus gallus histone cluster 1 H4-VI germinal H4 (similar to human histone  cluster 1 class H4 genes) (HIST1H46) mRNA. - RefSeq mRNA NM_001037845 |
| 2161,1185 | -21,62 | 60.1 (M:60.1) | Dimethyl: 1, 18 | 61 - 78 | ENSGALP00000041526 | Gallus gallus histone cluster 1 H4-VI germinal H4 (similar to human histone  cluster 1 class H4 genes) (HIST1H46) mRNA. - RefSeq mRNA NM_001037845 |
| 1534,9866 | 13,03 | 63.1 (M:63.1) | Dimethyl:2H(4): 1, 1, 13 | 80 - 92 | ENSGALP00000041526 | Gallus gallus histone cluster 1 H4-VI germinal H4 (similar to human histone  cluster 1 class H4 genes) (HIST1H46) mRNA. - RefSeq mRNA NM_001037845 |
| 1212,6723 | -4,08 | 52.2 (M:52.2) | Dimethyl:2H(4): 1 | 47 - 56 | ENSGALP00000041526 | Gallus gallus histone cluster 1 H4-VI germinal H4 (similar to human histone  cluster 1 class H4 genes) (HIST1H46) mRNA. - RefSeq mRNA NM_001037845 |
| 1017,5978 | -11,18 | 53.1 (M:53.1) | Dimethyl: 1 | 61 - 68 | ENSGALP00000041526 | Gallus gallus histone cluster 1 H4-VI germinal H4 (similar to human histone  cluster 1 class H4 genes) (HIST1H46) mRNA. - RefSeq mRNA NM_001037845 |
| 1358,7851 | 23,05 | 22.1 (M:22.1) | Dimethyl:2H(4): 12; Oxidation: 5 | 81 - 92 | ENSGALP00000041526 | Gallus gallus histone cluster 1 H4-VI germinal H4 (similar to human histone  cluster 1 class H4 genes) (HIST1H46) mRNA. - RefSeq mRNA NM_001037845 |
| 1382,7648 | 3,47 | 68.6 (M:68.6) | Oxidation: 5; Dimethyl: 1, 12 | 81 - 92 | ENSGALP00000041526 | Gallus gallus histone cluster 1 H4-VI germinal H4 (similar to human histone  cluster 1 class H4 genes) (HIST1H46) mRNA. - RefSeq mRNA NM_001037845 |
| 1366,7717 | 4,89 | 102.2 (M:102.2) | Dimethyl: 1, 12 | 81 - 92 | ENSGALP00000041526 | Gallus gallus histone cluster 1 H4-VI germinal H4 (similar to human histone  cluster 1 class H4 genes) (HIST1H46) mRNA. - RefSeq mRNA NM_001037845 |
| 1338,7392 | 4,1 | 15.1 (M:15.1) | Dimethyl: 1 | 81 - 92 | ENSGALP00000041526 | Gallus gallus histone cluster 1 H4-VI germinal H4 (similar to human histone  cluster 1 class H4 genes) (HIST1H46) mRNA. - RefSeq mRNA NM_001037845 |
| 1374,8211 | 4,21 | 85.4 (M:85.4) | Dimethyl:2H(4): 1, 12 | 81 - 92 | ENSGALP00000041526 | Gallus gallus histone cluster 1 H4-VI germinal H4 (similar to human histone  cluster 1 class H4 genes) (HIST1H46) mRNA. - RefSeq mRNA NM_001037845 |
| 1390,8094 | -0,56 | 50.9 (M:50.9) | Dimethyl:2H(4): 1, 12; Oxidation: 5 | 81 - 92 | ENSGALP00000041526 | Gallus gallus histone cluster 1 H4-VI germinal H4 (similar to human histone  cluster 1 class H4 genes) (HIST1H46) mRNA. - RefSeq mRNA NM_001037845 |
| 1526,8805 | -7,04 | 70.0 (M:70.0) | Dimethyl:2H(4): 1; Dimethyl: 12 | 81 - 93 | ENSGALP00000041526 | Gallus gallus histone cluster 1 H4-VI germinal H4 (similar to human histone  cluster 1 class H4 genes) (HIST1H46) mRNA. - RefSeq mRNA NM_001037845 |
| 1154,6456 | 9,16 | 28.8 (M:28.8) | Dimethyl: 1, 5 | 51 - 59 | ENSGALP00000008341 | glutathione S-transferase theta 1-like - CGNC Symbol 55583 |
| 726,446 | -6,66 | 22.6 (M:22.6) | Dimethyl: 1 | 243 - 248 | ENSGALP00000008341 | glutathione S-transferase theta 1-like - CGNC Symbol 55583 |
| 1353,7078 | 6,98 | 57.0 (M:57.0) | Carbamidomethyl: 1; Dimethyl: 1, 9 | 41 - 50 | ENSGALP00000008341 | glutathione S-transferase theta 1-like - CGNC Symbol 55583 |
| 875,5406 | 2,68 | 17.8 (M:17.8) | Oxidation: 6; Dimethyl: 1 | 65 - 71 | ENSGALP00000008341 | glutathione S-transferase theta 1-like - CGNC Symbol 55583 |
| 1845,9463 | -3,87 | 52.6 (M:52.6) | Dimethyl: 1, 2, 14 | 96 - 109 | ENSGALP00000008341 | glutathione S-transferase theta 1-like - CGNC Symbol 55583 |
| 867,506 | -3,11 | 34.0 (M:34.0) | Dimethyl: 1 | 34 - 40 | ENSGALP00000008341 | glutathione S-transferase theta 1-like - CGNC Symbol 55583 |
| 1672,8425 | -0,98 | 86.4 (M:86.4) | Dimethyl: 1 | 113 - 125 | ENSGALP00000008341 | glutathione S-transferase theta 1-like - CGNC Symbol 55583 |
| 819,4724 | -4,06 | 23.0 (M:23.0) | Oxidation: 5; Dimethyl: 6 | 50 - 55 | ENSGALP00000008341 | glutathione S-transferase theta 1-like - CGNC Symbol 55583 |
| 878,5535 | -3,99 | 56.5 (M:56.5) | Dimethyl: 1 | 83 - 89 | ENSGALP00000040653 | Gallus gallus H2A histone family member J (H2AFJ) mRNA. - RefSeq mRNA NM_001030753 |
| 976,587 | -0,7 | 60.6 (M:60.6) | Dimethyl:2H(4): 1 | 22 - 30 | ENSGALP00000040653 | Gallus gallus H2A histone family member J (H2AFJ) mRNA. - RefSeq mRNA NM_001030753 |
| 1748,9681 | 1,56 | 97.5 (M:97.5) | Dimethyl: 1, 14 | 83 - 96 | ENSGALP00000040653 | Gallus gallus H2A histone family member J (H2AFJ) mRNA. - RefSeq mRNA NM_001030753 |
| 972,554 | -8,77 | 78.8 (M:78.8) | Dimethyl: 1 | 22 - 30 | ENSGALP00000040653 | Gallus gallus H2A histone family member J (H2AFJ) mRNA. - RefSeq mRNA NM_001030753 |
| 1328,741 | -0,76 | 15.8 (M:15.8) | Dimethyl: 1, 11 | 90 - 100 | ENSGALP00000040653 | Gallus gallus H2A histone family member J (H2AFJ) mRNA. - RefSeq mRNA NM_001030753 |
| 1356,7725 | -0,58 | 64.5 (M:64.5) | Dimethyl: 1, 7, 11 | 90 - 100 | ENSGALP00000040653 | Gallus gallus H2A histone family member J (H2AFJ) mRNA. - RefSeq mRNA NM_001030753 |
| 1959,1606 | -20,19 | 29.6 (M:29.6) | Dimethyl: 19 | 101 - 119 | ENSGALP00000040653 | Gallus gallus H2A histone family member J (H2AFJ) mRNA. - RefSeq mRNA NM_001030753 |
| 1959,2055 | 2,73 | 37.7 (M:37.7) | Dimethyl: 1 | 101 - 119 | ENSGALP00000040653 | Gallus gallus H2A histone family member J (H2AFJ) mRNA. - RefSeq mRNA NM_001030753 |
| 1987,2365 | 2,55 | 131.8 (M:131.8) | Dimethyl: 1, 19 | 101 - 119 | ENSGALP00000040653 | Gallus gallus H2A histone family member J (H2AFJ) mRNA. - RefSeq mRNA NM_001030753 |
| 1995,2801 | -0,74 | 79.3 (M:79.3) | Dimethyl:2H(4): 1, 19 | 101 - 119 | ENSGALP00000040653 | Gallus gallus H2A histone family member J (H2AFJ) mRNA. - RefSeq mRNA NM_001030753 |
| 917,4529 | -4,96 | 27.2 (M:27.2) | Dimethyl: 1, 7 | 90 - 96 | ENSGALP00000040653 | Gallus gallus H2A histone family member J (H2AFJ) mRNA. - RefSeq mRNA NM_001030753 |
| 1188,6693 | 5,87 | 21.2 (M:21.2) | Dimethyl: 1, 6 | 77 - 86 | ENSGALP00000027541 | high mobility group box 1 - CGNC Symbol 49444 |
| 1000,5897 | 7,12 | 52.8 (M:52.8) | Dimethyl: 1, 2, 8 | 89 - 96 | ENSGALP00000027541 | high mobility group box 1 - CGNC Symbol 49444 |
| 1188,6681 | 4,87 | 31.3 (M:31.3) | Dimethyl: 1, 10 | 77 - 86 | ENSGALP00000027541 | high mobility group box 1 - CGNC Symbol 49444 |
| 1216,6988 | 4,27 | 72.5 (M:72.5) | Dimethyl: 1, 6, 10 | 77 - 86 | ENSGALP00000027541 | high mobility group box 1 - CGNC Symbol 49444 |
| 1228,7744 | 4,44 | 62.9 (M:62.9) | Dimethyl:2H(4): 1, 6, 10 | 77 - 86 | ENSGALP00000027541 | high mobility group box 1 - CGNC Symbol 49444 |
| 781,5042 | -2,09 | 27.0 (M:27.0) | Dimethyl:2H(4): 1, 6 | 77 - 82 | ENSGALP00000027541 | high mobility group box 1 - CGNC Symbol 49444 |
| 773,447 | -11,12 | 33.0 (M:33.0) | Dimethyl: 1, 6 | 77 - 82 | ENSGALP00000027541 | high mobility group box 1 - CGNC Symbol 49444 |
| 1009,5338 | -4,86 | 38.7 (M:38.7) | Dimethyl: 1, 2, 8 | 58 - 65 | ENSGALP00000027541 | high mobility group box 1 - CGNC Symbol 49444 |
| 1021,6082 | -5,68 | 17.5 (M:17.5) | Dimethyl:2H(4): 1, 2, 8 | 58 - 65 | ENSGALP00000027541 | high mobility group box 1 - CGNC Symbol 49444 |
| 1184,6322 | 0,97 | 71.3 (M:71.3) | Dimethyl: 1, 3 | 155 - 163 | ENSGALP00000027541 | high mobility group box 1 - CGNC Symbol 49444 |
| 1192,6835 | 1,92 | 16.0 (M:16.0) | Dimethyl:2H(4): 1, 3 | 155 - 163 | ENSGALP00000027541 | high mobility group box 1 - CGNC Symbol 49444 |
| 736,3915 | -9,97 | 28.1 (M:28.1) | Dimethyl: 1 | 158 - 163 | ENSGALP00000027541 | high mobility group box 1 - CGNC Symbol 49444 |
| 1604,9376 | 0,37 | 100.4 (M:100.4) | Dimethyl: 1, 2, 15 | 113 - 127 | ENSGALP00000027541 | high mobility group box 1 - CGNC Symbol 49444 |
| 1617,0143 | 1,23 | 119.8 (M:119.8) | Dimethyl:2H(4): 1, 2, 15 | 113 - 127 | ENSGALP00000027541 | high mobility group box 1 - CGNC Symbol 49444 |
| 1648,8143 | -11,3 | 33.0 (M:33.0) | Dimethyl: 1, 14 | 30 - 43 | ENSGALP00000027541 | high mobility group box 1 - CGNC Symbol 49444 |
| 1676,8394 | -14,85 | 51.0 (M:51.0) | Dimethyl: 1, 1, 14 | 30 - 43 | ENSGALP00000027541 | high mobility group box 1 - CGNC Symbol 49444 |
| 1684,8853 | -17,29 | 49.1 (M:49.1) | Dimethyl:2H(4): 1, 1; Dimethyl: 14 | 30 - 43 | ENSGALP00000027541 | high mobility group box 1 - CGNC Symbol 49444 |
| 1688,9343 | -3,11 | 60.0 (M:60.0) | Dimethyl:2H(4): 1, 1, 14 | 30 - 43 | ENSGALP00000027541 | high mobility group box 1 - CGNC Symbol 49444 |
| 1680,8918 | 1,45 | 30.7 (M:30.7) | Dimethyl:2H(4): 1; Dimethyl: 1, 14 | 30 - 43 | ENSGALP00000027541 | high mobility group box 1 - CGNC Symbol 49444 |
| 1520,71 | -18,41 | 37.6 (M:37.6) | Dimethyl: 1, 13 | 31 - 43 | ENSGALP00000027541 | high mobility group box 1 - CGNC Symbol 49444 |
| 1524,7319 | -20,44 | 36.0 (M:36.0) | Dimethyl:2H(4): 1; Dimethyl: 13 | 31 - 43 | ENSGALP00000027541 | high mobility group box 1 - CGNC Symbol 49444 |
| 1528,7813 | -4,5 | 56.4 (M:56.4) | Dimethyl:2H(4): 1, 13 | 31 - 43 | ENSGALP00000027541 | high mobility group box 1 - CGNC Symbol 49444 |
| 1544,7144 | -1,97 | 27.1 (M:27.1) | Carbamidomethyl: 11; Dimethyl:2H(4): 1; Oxidation: 1 | 13 - 24 | ENSGALP00000027541 | high mobility group box 1 - CGNC Symbol 49444 |
| 1220,6917 | -22,1 | 20.3 (M:20.3) | Dimethyl:2H(4): 6; Dimethyl: 1, 10 | 77 - 86 | ENSGALP00000027541 | high mobility group box 1 - CGNC Symbol 49444 |
| 1524,7112 | 9,06 | 88.8 (M:88.8) | Carbamidomethyl: 11; Dimethyl: 1 | 13 - 24 | ENSGALP00000027541 | high mobility group box 1 - CGNC Symbol 49444 |
| 1528,7379 | 10,11 | 71.5 (M:71.5) | Carbamidomethyl: 11; Dimethyl:2H(4): 1 | 13 - 24 | ENSGALP00000027541 | high mobility group box 1 - CGNC Symbol 49444 |
| 1540,6971 | 3,12 | 49.6 (M:49.6) | Carbamidomethyl: 11; Oxidation: 1; Dimethyl: 1 | 13 - 24 | ENSGALP00000027541 | high mobility group box 1 - CGNC Symbol 49444 |
